# Amiodarone or Implantable Cardioverter-Defibrillator in Chagas Cardiomyopathy: The CHAGASICS Randomized Clinical Trial

**DOI:** 10.1001/jamacardio.2024.3169

**Published:** 2024-10-02

**Authors:** Martino Martinelli-Filho, José A. Marin-Neto, Mauricio Ibrahim Scanavacca, Angelo Amato Vincenzo de Paola, Paulo de Tarso Jorge Medeiros, Ruth Owen, Stuart J. Pocock, Sergio Freitas de Siqueira

**Affiliations:** 1 Department of Cardiology, Instituto do Coração, Hospital das Clínicas da Universidade de São Paulo, São Paulo, Brazil; 2 Department of Interventional Cardiology, Faculdade de Medicina de Ribeirão Preto, Universidade de São Paulo, Ribeirão Preto, São Paulo, Brazil; 3 Department of Cardiology, Escola Paulista de Medicina, São Paulo, Brazil; 4 Department of Cardiology, Instituto Dante Pazzanese de Cardiologia, São Paulo, Brazil; 5 Department of Medical Statistics, London School of Hygiene and Tropical Medicine, London, United Kingdom; 6 Oxon Epidemiology, Madrid, Spain

## Abstract

**Importance:**

Over 10 000 people with Chagas disease experience sudden cardiac death (SCD) annually, mostly caused by ventricular fibrillation. Amiodarone hydrochloride and the implantable cardioverter-defibrillator (ICD) have been empirically used to prevent SCD in patients with chronic Chagas cardiomyopathy.

**Objective:**

To test the hypothesis that ICD is more effective than amiodarone therapy for primary prevention of all-cause mortality in patients with chronic Chagas cardiomyopathy and moderate to high mortality risk, assessed by the Rassi score.

**Design, Setting, and Participants:**

CHAGASICS is an open-label, randomized clinical trial. The study enrolled patients from 13 centers in Brazil from May 30, 2014, to August 13, 2021, with the last follow-up November 8, 2021. Patients with serological findings positive for Chagas disease, a Rassi risk score of at least 10 points (intermediate to high risk), and at least 1 episode of nonsustained ventricular tachycardia were eligible to participate. Data were analyzed from May 3, 2022, to June 16, 2023.

**Interventions:**

Patients were randomized 1:1 to receive ICD or amiodarone (with a loading dose of 600 mg after randomization).

**Main Outcomes and Measures:**

The primary outcome was all-cause mortality, and secondary outcomes included SCD, hospitalization for heart failure, and necessity of a pacemaker during the entire follow-up.

**Results:**

The study was stopped prematurely for administrative reasons, with 323 patients randomized (166 in the amiodarone group and 157 in the ICD group), rather than the intended 1100 patients. Analysis was by intention to treat at a median follow-up of 3.6 (IQR, 1.8-4.4) years. Mean (SD) age was 57.4 (9.8) years, 185 patients (57.3%) were male, and the mean (SD) left ventricular ejection fraction was 37.0% (11.6%). There were 60 deaths (38.2%) in the ICD arm and 64 (38.6%) in the amiodarone group (hazard ratio [HR], 0.86 [95% CI, 0.60-1.22]; *P* = .40). The rates of SCD (6 [3.8%] vs 23 [13.9%]; HR, 0.25 [95% CI, 0.10-0.61]; *P* = .001), bradycardia requiring pacing (3 [1.9%] vs 27 [16.3%]; HR, 0.10 [95% CI, 0.03-0.34]; *P* < .001), and heart failure hospitalization (14 [8.9%] vs 28 [16.9%]; HR, 0.46 [95% CI, 0.24-0.87]; *P* = .01) were lower in the ICD group compared with the amiodarone arm.

**Conclusions and Relevance:**

In patients with chronic Chagas cardiomyopathy at moderate to high risk of mortality, ICD did not reduce the risk of all-cause mortality. However, ICD significantly reduced the risk of SCD, pacing need, and heart failure hospitalization compared with amiodarone therapy. Further studies are warranted to confirm the evidence generated by this trial.

**Trial Registration:**

ClinicalTrials.gov Identifier: NCT01722942

## Key Points

**Question:**

Are implantable cardioverter-defibrillators (ICDs) more effective than amiodarone hydrochloride for primary prevention of all-cause mortality and sudden cardiac death (SCD) in patients with chronic Chagas cardiomyopathy at moderate to high mortality risk?

**Findings:**

This randomized clinical trial included 323 patients, with 157 in the ICD group and 166 in the amiodarone arm. Treatment with ICD did not reduce the primary end point of all-cause mortality but significantly reduced SCD by 72% and heart failure hospitalization by 47%.

**Meaning:**

Compared with amiodarone, ICD did not reduce the risk of all-cause mortality but reduced the risk of SCD and heart failure; further studies are needed to confirm these results.

## Teaser

This randomized clinical trial assesses whether cardioverter-defibrillator implantation is more effective than amiodarone therapy for the primary prevention of all-cause mortality and secondary prevention of sudden cardiac death, hospitalization for heart failure, and use of a pacemaker among patients with chronic Chagas cardiomyopathy.

## Introduction

For many decades, Chagas disease has been a major public health problem in Latin America, where 75 million people are at risk of *Trypanosoma cruzi* infection and 6 to 7 million people are affected by the disease.^1^ More recently, migration, and globalization have resulted in a significant increase in the prevalence of Chagas disease worldwide, particularly in the US and Europe.^2^ In about 40% of patients with Chagas disease, the heart is affected, causing chronic Chagas cardiomyopathy (CCC), which leads to inflammation, cell death, and fibrosis. These cause wall motion abnormalities, ventricular aneurysm, heart failure (HF), thromboembolic events, and arrhythmia. Chronic Chagas cardiomyopathy is an essentially arrhythmogenic disease, with a high prevalence of complex ventricular arrhythmias, including nonsustained ventricular tachycardia (NSVT), sustained VT, and ventricular fibrillation (VF), which is the leading cause of sudden cardiac death (SCD).^3,4^ Estimated annual deaths due to Chagas disease total 12 000, with 55% to 65% being SCD.^5^ Less often, a bradyarrhythmia, pulseless electrical activity, intractable HF, thromboembolic complications, or spontaneous ventricular rupture of an apical aneurysm may be the cause of death.^6^ The risk stratification of mortality in CCC can be established using the Rassi score, which includes the following factors: New York Heart Association (NYHA) functional classes III to IV, cardiomegaly, global and/or segmental ventricular dyssynergy on echocardiography, NSVT on Holter monitoring, low voltage on electrocardiography, and male sex.^4^ More recently, myocardial fibrosis evaluated by cardiac magnetic resonance imaging has also been associated with mortality in CCC.^7,8,9,10^ Several variables, such as sustained and nonsustained VT, right ventricular systolic dysfunction, late potentials on signal-averaged electrocardiography, QT-interval dispersion, and increased levels of brain natriuretic peptides, have been suggested in various studies as additional factors associated with bad prognosis and augmented risk of mortality in CCC, but these investigations lacked an independent and extensive external validation.^4,8,11^ Prevention of SCD in patients with CCC has been attempted empirically with amiodarone hydrochloride, implantable cardioverter-defibrillators (ICDs), or their combination.^12,13,14,15,16,17^ No randomized clinical trial (RCT), to our knowledge, has evaluated the efficacy of these therapies in this population.^5^ The ICD indication in patients with CCC is based on the extrapolation of RCTs showing ICD benefits in ischemic cardiomyopathy (ICM) and other causes of nonischemic cardiomyopathy (NICM).^18,19,20,21,22^ Therefore, the CHAGASICS (Chronic Use of Amiodarone Against Implantable Cardioverter-Defibrillator Therapy for Primary Prevention of Death in Patients With Chagas Cardiomyopathy Study) trial tested the hypothesis that an ICD is more effective than amiodarone therapy for the primary prevention of all-cause mortality in patients with CCC and moderate to high mortality risk as assessed with the Rassi score.^4^

## Methods

### Study Design

**Figure 1.**
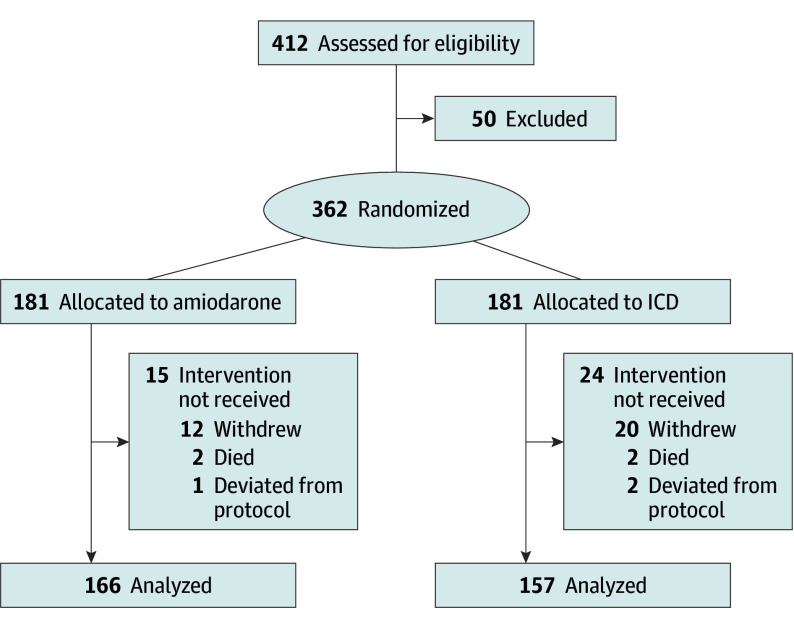
CONSORT Flowchart ICD indicates implantable cardioverter defibrillator.

CHAGASICS is an open-label, parallel-group, RCT conducted in at 13 centers in Brazil and designed to assess whether ICDs are more effective than amiodarone therapy for the primary prevention of all-cause mortality (Figure 1). CHAGASICS adhered fully to the ethical principles of the Declaration of Helsinki,^23^ the specifications of the International Conference on Harmonization Good Clinical Practice guidelines,^24^ and followed the Consolidated Standards of Reporting Trials (CONSORT) reporting guideline. The trial was approved by each center’s research ethics committee, and every patient who agreed to participate in the trial provided written informed consent. The rationale and design of the study were previously published.^25^ The trial protocol is found in Supplement 1.

### Participants

Patients from 13 centers in Brazil participated in the study, with the Heart Institute of Hospital das Clínicas of the Medical School of the University of São Paulo being the core data coordinating center. Patients were eligible for the study if they met the following inclusion criteria: 18 to 75 years of age; documented positive serological test result for Chagas disease by at least 2 different methods (indirect hemagglutination, indirect immunofluorescence, or enzyme-linked immunosorbent assay) in the past 6 months; Rassi risk score at least 10 points; and at least 1 documented episode of NSVT on 24-hour Holter monitoring, which is defined as at least 3 successive ventricular ectopic beats (duration up to 30 seconds), with a heart rate of greater than 120 beats/min.^4^

### Outcomes

The primary outcome was all-cause mortality. Local clinical investigators were responsible for (1) finding out whether patients died and immediately reporting deaths to the data coordination center, (2) verifying whether patients who did not attend scheduled follow-up appointments were alive, and (3) categorizing the causes of death based on available information from witnesses, relatives and family members, death certificates, hospital records, autopsy reports, and device evaluation where applicable. The categories of cause of death were SCD, HF death, other causes of cardiovascular death, noncardiovascular death, and unknown. The secondary outcomes were SCD, cardiovascular death, HF death and hospitalization, and the need for pacemaker implantation or ICD pacing in cases of severe bradyarrhythmia.

### Randomization and Follow-Up

The randomization sequence was computer generated, and allocation concealment was ensured. Eligible patients were randomly assigned 1:1 to receive an ICD or amiodarone immediately after the baseline examination.

The follow-up visits were scheduled at 10 days, at 1 and 4 months, and at 4-month intervals for up to 6 years after therapy initiation. The protocol of visits has been previously described.^25^ The duration of follow-up was from the time of randomization until death, the study termination date, or censorship after loss to follow-up.

### ICD and Amiodarone Use

Patients assigned to the ICD group had the device implanted as soon as possible after randomization. For patients in this group, the study encouraged the local clinician investigator to (1) choose, preferentially, a single-chamber device; (2) program the ICD to back up pacing in a ventricular-inhibited mode at a rate of 40 beats/min, to detect VF at 180 beats/min, and to provide shock with maximum energy; (3) activate the antitachycardia pacing to deliver 8-beat bursts that begin at 81% of the tachycardia cycle duration for those cycles below the VF threshold and repeating this strategy if necessary; and (4) avoid the use of amiodarone, except in cases of multiple ICD shocks and electrical storm, in those with CCC refractory to β-blockers (including sotalol hydrochloride), catheter ablation, or both.

Patients assigned to the amiodarone group received amiodarone once a day. For this, the study encouraged the local clinical investigator to (1) initiate an oral loading dose of 600 mg/d for 10 days on an outpatient basis; (2) continue amiodarone use with an oral maintenance single dose of 200 to 400 mg/d until the end of the study; (3) consider the optimum maintenance dose for each patient (investigator discretion) based on antiarrhythmic efficacy on 24-hour Holter monitor, resting heart rate, adverse effects, and an excessively prolonged corrected QT interval; (4) adjust doses during follow-up, aiming at a maintenance dose of 200 to 400 mg/d; (5) recommend pacemaker implantation in cases of severe bradyarrhythmia for supporting appropriate amiodarone use; and (6) indicate an ICD should any episode of sustained VT or VF occur during follow-up.

Although treatment crossover in either direction was strongly discouraged, it was permitted when it was clearly in the patient’s best interest. Concerning concomitant medical therapy, for both groups, local clinician investigators were encouraged to (1) optimize the use of angiotensin-converting enzyme inhibitors (or angiotensin II receptor blockers), spironolactone, diuretics, and oral anticoagulants (when appropriate); and (2) not introduce any other antiarrhythmic drugs, except sotalol.

### Statistical Analysis

The intended trial size of 1100 patients, followed up for a mean 4.5 years, was based on having 90% power to detect a 30% relative reduction in all-cause mortality with ICD, assuming a 30% death rate in the control group receiving amiodarone with 2-sided type I error of 0.05, allowing for 10% loss to follow-up. The final sample size was 362 patients with a median follow-up of 3.6 (IQR, 1.8-4.4) years.

Analyses of the primary end point all-cause death used a Cox proportional hazards model,^26^ generating a hazard ratio (HR) with 95% CI and log-rank *P* value. The proportional hazards assumption was assessed visually using log-log transformed Kaplan-Meier plots and Schoenfeld tests. If violated, an additional restricted mean survival time (RMST) analysis was performed. Kaplan-Meier plots by treatment group display the time pattern of any treatment differences.^27^ Similar analyses were performed for secondary cause–specific mortality findings and for time to first HF hospitalization.

The incidence of bradycardia requiring a pacemaker implantation (amiodarone arm), or pacing (ICD arm) was a comparison of percentage rates by treatment group, leading to an odds ratio with 95% CI and a Fisher exact test *P* value. Change in left ventricular ejection fraction (LVEF) over follow-up was assessed using analysis of covariance adjusted for baseline value. The NYHA functional class during follow-up with death categorized as the worst outcome was compared between treatment groups using ordinal logistic regression. Prespecified subgroup analyses for all-cause death and for SCD compared HRs by subgroup using statistical tests of interaction.^28^

All analyses were based on a modified intention-to-treat population, whereby patients who did not receive their randomized treatment were excluded.^29^ All analyses were performed using Stata, version 17.0 (StataCorp LLC). Two-sided *P* < .05 indicated statistical significance. Data were analyzed from May 3, 2022, to June 16, 2023.

## Results

### Study Population

The study was stopped prematurely due to the difficulty of enrolling patients during the COVID-19 pandemic and cessation of financial support. From May 30, 2014, to August 13, 2021, 362 patients were randomized at 13 centers, including 181 randomized to ICD and 181 to amiodarone (Figure 1). Four patients died (2 in each arm) and 35 were withdrawn for various reasons before initial therapy. Of the remaining 323 patients, 166 were in the amiodarone group and 157 in the ICD group (Figure 1). Among them, 284 patients were included until 2017, and 39 between 2018 and 2021. Follow-up was completed November 8, 2021.

Overall, the 2 groups were well balanced regarding baseline characteristics (Table 1). Mean (SD) age was 57.4 (9.8) years; 185 (57.3%) were male and 138 (42.7%) were female; mean (SD) LVEF was 37.0% (11.6%); and 206 (63.8%) were classified as being at high risk for mortality (Table 1). The target dose of amiodarone was achieved in most of the patients during the follow-up period (eTable 1 in Supplement 2).

**Table 1.** Baseline Characteristics of the Analyzed Population

Characteristic	Patients, No (%)	
	ICD (n = 157)	Amiodarone (n = 166)
Age, mean (SD), y	57.6 (9.7)	57.3 (9.9)
Sex		
Female	61 (38.9)	77 (46.4)
Male	96 (61.1)	89 (53.6)
BMI, mean (SD)	25.6 (4.1)	26.2 (5.1)
NYHA functional class		
I	51 (32.5)	47 (28.3)
II	65 (41.4)	77 (46.4)
III	38 (24.2)	39 (23.5)
IV	1 (0.6)	0
NA	2 (1.3)	3 (1.8)
Rassi risk strata		
Intermediate risk (10 to 11)	50 (31.8)	67 (40.4)
High risk (12 to 20)	107 (68.2)	99 (59.6)
LVEF, mean (SD), %	37.1 (10.9)	38.1 (11.1)
Medications used at baseline		
ACEI	77 (49.0)	74 (44.6)
ARB	40 (25.5)	37 (22.3)
β-Blocker	115 (73.2)	112 (67.5)
Calcium channel blocker	4 (2.5)	7 (4.2)
Digitalis	5 (3.2)	12 (7.2)
Diuretic	100 (63.7)	93 (56.0)
Nitrate	3 (1.9)	6 (3.6)
Hydralazine	4 (2.5)	13 (7.8)
Other antiarrhythmic	1 (0.6)	0
Other	110 (70.1)	122 (73.5)

Abbreviations: ACEI, angiotensin-converting enzyme inhibitor; ARB, angiotensin receptor blocker; BMI, body mass index (calculated as the weight in kilograms divided by the height in meters squared); ICD, implantable cardioverter-defibrillator; LVEF, left ventricle ejection fraction; NA, not applicable; NYHA, New York Heart Association.

### Primary and Secondary Outcomes

**Figure 2.**
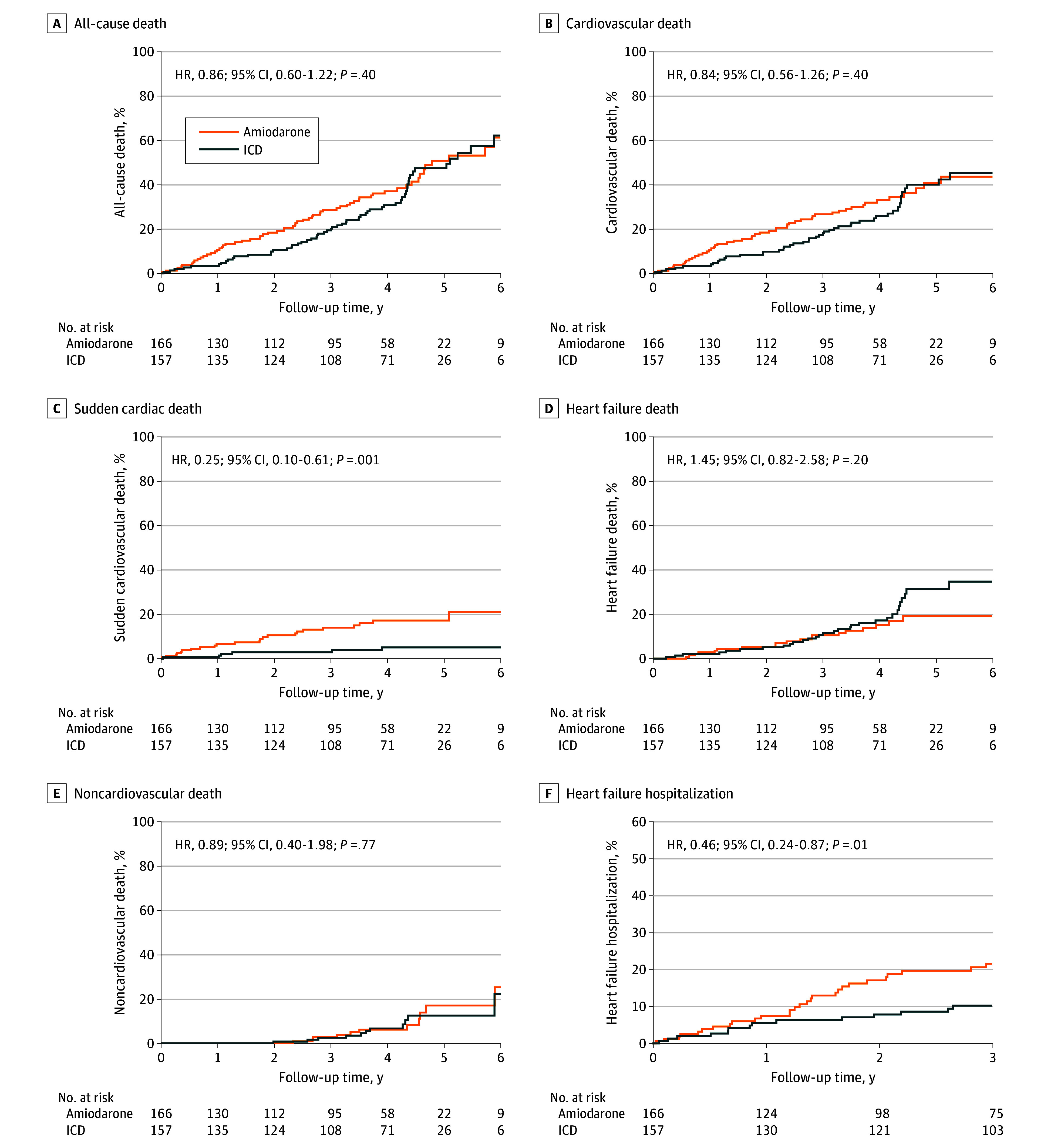
Cumulative Incidence for Primary and Secondary Study Outcomes HR indicates hazard ratio; ICD, implantable cardioverter defibrillator.

The median follow-up period was 3.6 (IQR, 1.8-4.4) years. The primary outcome, all-cause death, occurred in 60 patients in the ICD group (38.2%) and 64 (38.6%) in the amiodarone group (HR, 0.86 [95% CI, 0.60-1.22]; *P* = .40). Causes of death are shown in Table 2. In each group, 2 deaths were of unknown cause. Nonproportional risks for all causes of death are evident in Figure 2A. Hence, an RMST analysis up to the 5-year follow-up was performed. This showed a mean treatment difference in time alive of 104 days with ICD, but with a large 95% CI of −22 to 229 days (*P* = .10) (eTable 2 in Supplement 2). There was an early reduction in all-cause mortality with ICD (32% relative reduction at 3 years), which decreased to 17% at 4 years and 7% at 5 years (Figure 2A). Sudden cardiac death occurred in 6 patients (3.8%) in the ICD group and 23 patients (13.9%) in the amiodarone group, indicating reduction by 72% (HR, 0.25 [95% CI, 0.10-0.61]; *P* = .001) (Figure 2C and Table 2).

**Table 2.** Findings of Primary and Secondary End Points of the Study

End point	Study group, No. (%)		HR (95% CI)	*P* value^a^
	ICD (n = 157)	Amiodarone (n = 166)		
Primary				
All-cause death	60 (38.2)	64 (38.6)	0.86 (0.60-1.22)	.40
Secondary				
Cardiovascular death	46 (29.3)	50 (30.1)	0.84 (0.56-1.26)	.39
Sudden cardiac death	6 (3.8)	23 (13.9)	0.25 (0.10-0.61)	.001
Heart failure death	31 (19.7)	19 (11.4)	1.45 (0.82-2.58)	.20
Noncardiovascular death	12 (7.6)	12 (7.2)	0.89 (0.40-1.98)	.77
Heart failure hospitalization^b^	14 (8.9)	28 (16.9)	0.46 (0.24-0.87)	.01
Bradycardia requiring pacemaker	3 (1.9)	27 (16.3)	0.10 (0.03-0.34)^c^	<.001

Abbreviations: HR, hazard ratio; ICD, implantable cardioverter–defibrillator.

^a^ Unless otherwise indicated, calculated using a log-rank test.

^b^ Data on hospitalizations were only collected during 3 years of follow-up.

^c^ Reported as odds ratio (95% CI), with Fisher exact test *P* value.

There was no difference between the ICD and amiodarone groups for cardiovascular death (46 [29.3%] vs 50 [30.1%]; HR, 0.84 [95% CI, 0.56-1.26]; *P* = .39; RMST difference, 104 [95% CI, −22 to 229] days; *P* = .11) and HF death (31 [19.7%] vs 19 [11.4%]; HR, 1.45 [95% CI, 0.82-2.58]; *P* = .20) as shown in Figure 2B and D, Table 2, and eTable 2 in Supplement 2.

Hospitalization for HF occurred in 14 patients (8.9%) in the ICD group and 28 (16.9%) in the amiodarone group (HR, 0.46 [95% CI, 0.24-0.87]; *P* = .01; RMST difference, 22 [95% CI, 18-277] days; *P* = .03) (Figure 2F, Table 2, and eTable 2 in Supplement 2). The incidence of bradycardia warranting pacemaker implantation or pacing with a previously inserted device was 3 patients (1.9%) in the ICD group vs 27 (16.3%) in the amiodarone group, a decrease of 89% (HR, 0.10 [95% CI, 0.03-0.34]; *P* < .001) (Table 2).

### Subgroup Analysis

**Figure 3.**
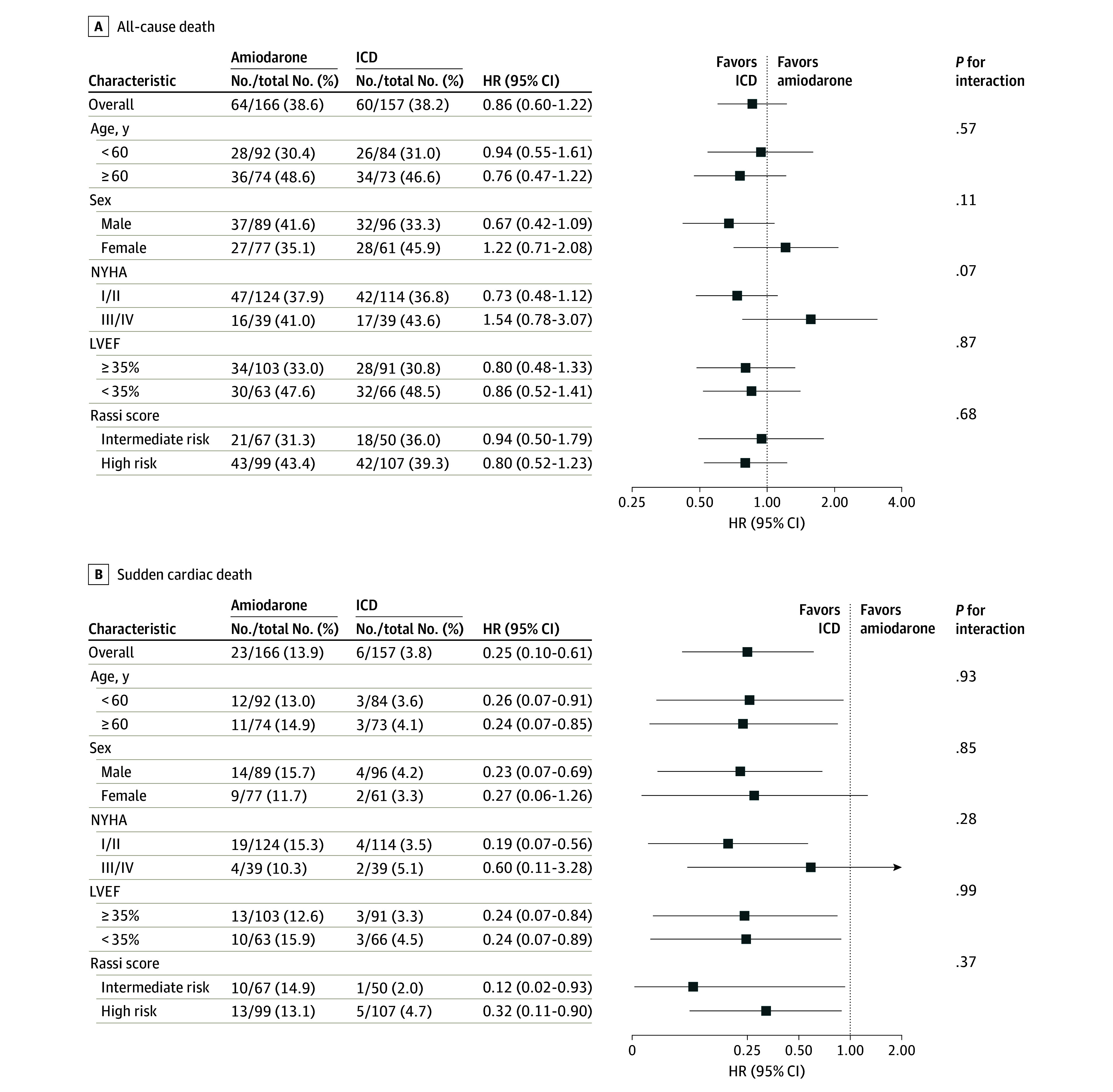
Subgroup Analysis of the Hazard Risks for All-Cause Death and Sudden Cardiac Death HR indicates hazard ratio; ICD, implantable cardioverter defibrillator; LVEF, left ventricular ejection fraction; and NYHA, New York Heart Association functional class.

The analysis of subgroups of patients according to age, sex, clinical and functional baseline characteristics for all-cause mortality did not show any statistically significant interactions (Figure 3A). The reduced incidence of SCD with ICD compared with amiodarone was consistent across subgroups analyzed (Figure 3B).

### Additional Analyses

Regarding ICD interventions, antitachycardia pacing therapy occurred in 57 patients (36.3%). Of these, 50 received therapies classified as appropriate (ICD therapy delivered for rhythms considered to be VT) and 16 received therapies classified as inappropriate (ICD therapy delivered for rhythms not considered to be VT) (there was overlap of appropriate and inappropriate therapies for some patients). Shock therapy occurred in 56 (35.7%) patients, classified as appropriate in 40 and as inappropriate in 13 (eTable 3 in Supplement 1).

There was no difference in the change in LVEF over follow-up between the 2 groups (eTable 4 in Supplement 2). However, patients in the ICD group were more likely to have an improved NYHA functional class over follow-up (common odds ratio at 3 years, 0.57 [95% CI, 0.37-0.89]; *P* = .01) (eTable 5 in Supplement 2).

Adverse events occurred in 14 patients (4.3%), including 10 (3.1%) in the amiodarone arm. In the ICD arm, most adverse events were related to lead dysfunction, and in the amiodarone arm they were mostly due to iatrogenically induced hypothyroidism (eTable 6 in Supplement 2). There were no significant differences in concomitant medical therapies for heart failure between the 2 groups during the follow-up period (eTable 7 in Supplement 2).

## Discussion

To our knowledge, CHAGASICS is the first RCT designed to assess whether ICD implantation is more effective than amiodarone therapy for the primary prevention of death in patients with CCC and moderate and high mortality risk. Despite premature interruption of the study due to low enrollment, relevant new data were obtained. Cardioverter-defibrillator implantation did not decrease the primary end point of all-cause mortality, but ICD did reduce several secondary end points, including the risk of SCD and HF hospitalization and the need for cardiac pacing.

The results of the present study show no reduction in overall mortality by ICD for primary prevention in CCC, in contrast with findings of studies of patients with ICM.^30,31,32^ The MUSTT (Multicenter Unsustained Tachycardia Trial)^30^ and MADIT (Multicenter Automatic Defibrillator Implantation Trial) I and II^31,32^ reported reduction in all-cause mortality with ICD treatment, ranging from 28.3% to 40.7%, compared with medical therapy. However, our results are in agreement with the RCTs for primary prevention in patients with NICM (DEFINITE [Defibrillators in Non-Ischemic Cardiomyopathy Treatment Evaluation]^33^ and DANISH [Danish ICD Study in Patients With Dilated Cardiomyopathy]^34^), which failed to demonstrate a reduction in all-cause mortality by ICD but reported, similarly to CHAGASICS, a significant relative risk reduction of SCD of 54% and 48%, respectively. Although 2 systematic reviews and meta-analyses comparing ICD with medical therapy for the primary prevention of death in patients with NICM reported reduction of SCD rates with ICD,^35,36^ only one of these studies reported a reduction in all-cause mortality.

As for the more general indications of either amiodarone or ICD treatment in patients with CCC for the primary prevention of death, recommendations are extrapolated from studies performed in patients with ICM and NICM.^5^ According to meta-analyses referring to primary prevention, with 18 studies of low-quality evidence, amiodarone showed a modest but significant reduction in SCD rate compared with placebo.^37^ On the other hand, SCD-HEFT (Sudden Cardiac Death in Heart Failure Trial) was the only RCT of primary prevention of death showing ICD benefits over medical therapy in patients with NICM.^21^ The study reported a 23% mortality reduction (at 4.5 months of follow-up) by ICD compared with placebo and no favorable effect of amiodarone on survival. After an 11-year follow-up, the ICD benefit persisted with a mortality reduction of 13% (*P* = .03).^22^ However, the direct extrapolation of results of SCD-HEFT to the context of CCC is hindered because it showed ICD benefit only in patients in NYHA class II.^22^

It is noteworthy that the failure of ICD in our trial to reduce all-cause mortality as a primary prevention strategy corroborates the lack of benefit even for secondary prevention of all-cause death in patients with CCC.^5^ A systematic review and meta-analysis of 13 retrospective studies including 1041 patients^38^ suggested that ICD implantation, compared with amiodarone, was not associated with a lower all-cause mortality rate. The reported annual mortality rate was 9.7% (95% CI, 5.7%-13.7%) for ICD and 9.6% (95% CI, 6.7%-12.4%) (*P* = .95) for amiodarone.

It is important to point out that based on the evidence discussed above, mostly gathered through amiodarone’s widely empirical use for decades in South America, before the ICD era, not including amiodarone in the medical therapy of patients with CCC and a high risk for mortality is currently considered unreasonable. That is why we designed the CHAGASICS trial without a control group but with medical therapy only.^39,40,41^

In addition to effects on mortality, as described above, 2 other changes were detected in risk reduction with ICD implantation in CHAGASICS: HF hospitalization decreased by 47% (8.9% vs 16.9%; *P* = .01) and the necessity of pacing decreased by 89% (1.9% vs 16.3%; *P* < .001). These changes could not be ascribed to differences in the use of concomitant medications. In our study, use of concomitant medical therapy, mostly aiming at control of HF, was well balanced between the study groups. The percentage of β-blocker use was lower than usually reported in patients with other cardiomyopathies, but similar to that in observational studies of patients with CCC.^42^ Therefore, the CHAGASICS protocol instructed the investigators to carefully weigh the use of β-blockers due to increased risk of bradyarrhythmias with the concomitant use of amiodarone. In fact, in the amiodarone arm, a greater need for pacing was observed. Furthermore, it is known that right ventricular stimulation applied to patients with ventricular dysfunction leads to worsening of HF, and it could explain the higher rate of HF hospitalizations observed in the amiodarone arm of our study.

The all-cause mortality rate in the present study was comparable to that reported by Rassi et al^4^ in a historical series of 424 patients with CCC. In patients with CCC at high risk of death studied before the ICD era, the annual all-cause mortality rate after 5-year follow-up was 12.2%. In our study, 63.8% of the patients had a high mortality risk and the annual all-cause mortality rate was very similar in both arms (12.9% for ICD and 12.7% for amiodarone). Similarly, in MADIT II, which included patients with ICM and LVEF of less than 35%, the all-cause mortality rate in the medical therapy arm was 11.9%. However, in MADIT II, the reduction in the annual all-cause mortality rate after 4 years was 28%, whereas in our study the reduction was 17%. It is plausible that in CCC a more aggressive remodeling process unchained by the incessant pathogenesis of myocardial inflammatory changes could limit the long-term benefit of ICD.^5,32^

The SCD-HEFT long-term follow-up analysis reported that the ICD benefit over medical therapy is time dependent. It persisted for 5 years with an all-cause mortality relative reduction of 28% (HR, 0.72 [95% CI, 0.55-0.96]), but from 6 years until the end of follow-up (9 years) there was no benefit from the ICD (HR, 0.82 [95% CI, 0.63-1.06]).^22^ In CHAGASICS, we observed the same scenario; all-cause mortality was 32% lower in the ICD group at 3 years, 17% lower at 4 years, and 7% lower at 5 years of follow-up.

This can possibly be explained by the fact that VT and VF occurred mainly in the first 4 years of follow-up, when the ICD was effective in reducing mortality. After 4 years, the mode of death in the ICD group became predominantly HF worsening, clearly shown in Figure 2D. These data agree with the findings of the REMADHE (Repetitive Education and Monitoring for Adherence for Heart Failure) study, which demonstrated that in patients with CCC and severe ventricular dysfunction, the predominant mode of death was HF.^43^

### Limitations

This study has some limitations. CHAGASIC recruited fewer patients (n = 362) than the 1100 initially intended, creating uncertainty regarding the conclusions, which should be interpreted cautiously. Specifically, the number of SCD events was small. Further, more patients withdrew after randomization in the ICD group, which could introduce bias. Also, there was no real control group, which precludes interpretation that either of the interventions would reduce overall mortality. We acknowledge that despite explicit protocol instructions regarding medical therapy, a low use of angiotensin-converting enzyme inhibitors, angiotensin II receptor blockers, and β-blockers may have influenced HF death and hospitalization end points (eTable 7 in Supplement 2). Another limitation is lack of a centralized clinical events committee, and causes of death were site determined. Heart failure hospitalization data were only available for the 3-year follow-up. Finally, patients requiring pacemaker implantation for bradycardia could not benefit from current conduction system stimulation, because these methods became widely available only after the implementation of CHAGASICS.

## Conclusions

This RCT showed that in patients with CCC at moderate to high risk of mortality, ICD compared with amiodarone did not reduce the risk of all-cause mortality. However, ICD significantly reduced several secondary end points, including the risk of SCD. Further studies are warranted to confirm the evidence generated by this trial.

## Notes

Supplement 1.
Trial Protocol

Supplement 2. **eTable 1.** Amiodarone Use During Follow-Up **eTable 2.** Findings of Primary and Secondary End Points of the Study **eTable 3.** Appropriate and Inappropriate ICD Therapies **eTable 4.** Change in Left Ventricular Ejection Fraction During Follow-Up **eTable 5.** NYHA Functional Class During Follow-Up **eTable 6.** Adverse Events by Treatment Group **eTable 7.** Concomitant Use of Medication at the End of Follow-Up by Treatment Group

Supplement 3. **Nonauthor Collaborators.** The CHAGASICS investigators

Supplement 4.
Data Sharing Statement
