# Supplementary material for: Amiodarone or Implantable Cardioverter-Defibrillator in Chagas Cardiomyopathy: The CHAGASICS Randomized Clinical Trial
Source: JAMA Cardiol. 2024 Oct 2;9(12):1073–81. doi: 10.1001/jamacardio.2024.3169 (PMC11447631; doi:10.1001/jamacardio.2024.3169)
Supplement: Supplement 1. — Trial Protocol [file jamacardiol-e243169-s001.pdf]

CHAGASICS: Amiodarone or ICD in Chagas Cardiomyopathy

Supplement

This supplement contains the following items:

Final protocol, sample size calculation and statistical analyses

Summary of protocol changes

Publication of the rationale of the study

CHronic use of **A**miodarone a**GA**in**S**t **I**mplantable Cardioverter Defibrillator therapy in **C**hagas' cardiomyopathy for Primary Prevention of Death. A Randomized Clinical **S**tudy.

Ensaio Clínico Randomizado de Prevenção Primária de Morte Na Cardiopatia Chagásica Crônica: Uso de Amiodarona Versus Cardiodesfibrilador Implantável

This document is confidential and property of the authors. Information contained in this document that has not yet been published shall not be disclosed without previous written authorization of the authors. Access to this document will necessarily be restricted to the relevant parties.

Índice

Introduction 5

The Chagas disease 5

The severity of the disease 5

Mechanisms of sudden cardiac death 8

Risk Stratification 9

Antiarrhythmic therapeutic options 10

Summary and Hypothesis 14

Objectives 15

Primary Objective 15

Secondary Objectives 15

Outcome variables 15

Primary Endpoint 15

Secondary Endpoints 15

Methods 16

Population Sample 16

Inclusion 16

Exclusion 16

Study Flowchart and Steps 17

Allocation List Randomization and confidentiality 18

ICD Group 19

Amiodarone Group 19

Clinical Follow-up 20

Unscheduled visits 20

Deaths Classification 20

Heart failure hospitalization 20

Statistical Plan and Sample Size Calculation 21

Statistical methods 22

Planned subgroup analysis 22

Planned sub-study 22

Special Considerations 23
Criteria for Discontinuation of Amiodarone 24
Definitive Interruption 24
Discontinuation at Investigator/Patient discretion or dose reduction 25
Notes on interruption 25
Adverse Events 25
Serious Adverse Events 26
Protocol Deviations 26
Risks and Benefits 27
Risks to the Patient 27
Patient Benefits 27
Study Organization 28
Ethical and Regulatory Basis and Data Quality Control 29
Timeline 30
Budget 30
Bibliographic references 32

Introduction

The Chagas disease

Chagas disease (Trypanosomiasis americana) is caused by the protozoan parasite Trypanosoma cruzi, transmitted to humans through the feces of a hematophagous insect, of the Triatominae family, in most cases[1]. Infection usually occurs in childhood and the acute phase has an incubation period of 1 to 2 weeks and can last up to 3 months. The chronic phase follows, in which for a long time - 2-4 decades in most cases - patients have only positive serology for Chagas disease (CD), without symptoms or other signs of clinically apparent disease.[2,3]

Therefore, such patients present the so-called indeterminate form of CD, whose prognosis is essentially benign.[4,5]

While, due to pathogenic mechanisms that are still incompletely understood[6], many patients remain with this form of the disease for life, about 30 to 50% of infected individuals progress to certain forms: cardiac, digestive, or mixed. Chronic Chagas Cardiomyopathy (CCC) with a hemodynamic pattern similar to that of idiopathic dilated cardiomyopathy, but with very marked pathophysiological peculiarities, is the most common and severe clinical form of CD, being responsible for significant morbidity and mortality in many Latin America countries (and with epidemiological relevance also contemporaneously recognized in countries with significant immigration by individuals from those countries).[7]

It is estimated that 8-10 million people are infected with Trypanosoma cruzi in Latin America and other countries.[8,9,10] Considering the worst-case scenario, based on the above estimates, it can be deduced that 3-5 million infected individuals will manifest clinical forms of the disease in its chronic phase.

The severity of the disease

The mortality rate among CD patients remains high in many Latin American countries and is strongly related to the presence of chronic cardiomyopathy. Currently, the average annual mortality rate for the infected population with

cardiac manifestations of CD is estimated to be of the order of 4%, ranging from 1 to 10% according to risk stratification based on clinical characteristics and simple cardiological tests[11] (see Tables 1 and 2 below).

Table 1. Independent variables of mortality in chronic Chagas' cardiomyopathy and their weight value

Risk factor	Score
NYHA Functional Class III or IV	5
Cardiomegaly (cardiothoracic index >0.50 on chest X-ray)	5
Abnormality of a segmental or global movement of the left ventricular wall (2D Echo)	3
Non-sustained VT (24h Holter monitoring)	3
Low voltage QRS (ECG)	2
Male gender	2

NYHA, New York Heart Association; VT, ventricular tachyarrhythmia; 2D Echo, two-dimensional echocardiogram; ECG, electrocardiogram.
Adapted from Reference 11.

Table 2. Risk score totalization for predicting mortality in chronic Chagas cardiomyopathy

Total score	All-cause mortality		Risk
	5 years	10 years	
0–6	2%	10%	Low
7–11	18%	44%	Intermediate
12–20	63%	84%	High

Adapted from Reference 11.

In addition to the criteria used in the risk stratification shown in Tables 1 and 2, several markers of worse prognosis have been identified by several authors,

especially with regard to sudden cardiac death in different clinical contexts[12,13,14,15,16,17,18,19,20,21,22,23,24,25,26,27,28,29,30,31].

Characteristics such as presyncope or syncope, left ventricular dysfunction and heart failure, sustained or non-sustained ventricular tachyarrhythmia (VT), severe bradyarrhythmia (sinus node disease and advanced atrioventricular block), and previous cardiac arrest were identified as markers of risk of sudden cardiac death (SCD). On the other hand, isolated premature ventricular contraction (PVC) on 24-h Holter monitoring and right bundle-branch block do not significantly interfere in the prognosis of CCC.

SCD is often associated with manifestations of heart failure (HF), but it can also occur in patients with asymptomatic left ventricular dysfunction[32,33,34]. It is responsible for approximately 55 to 65% of all causes of death, while refractory HF is a cause of death in about 25 to 30% of patients and systemic or pulmonary thromboembolism in about 10 to 15%[3,34,35,36,37]. Very rarely, aneurysm rupture can be the mechanism of sudden death in CCC[38].

Recently, the correlation between stages of CCC and causes of mortality was schematically described (Figure 1)[10]. SCD usually affects patients from stage II of the disease onwards, being more relevant in stage III and a little less in stage IV, in which refractory HF becomes quite frequent as a cause of death.

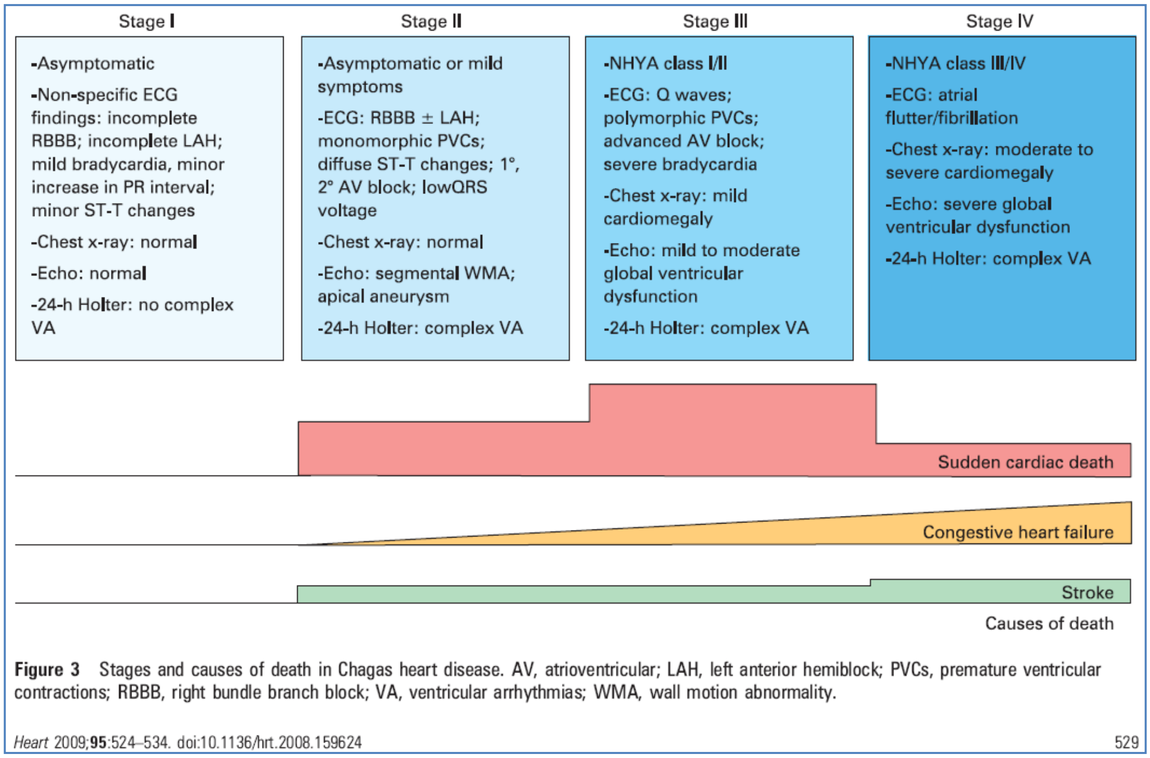

Figure 1. Relationship between the evolutionary stages of chronic Chagas cardiomyopathy and causes of death – Copied from Reference 10.

Mechanisms of sudden cardiac death

The main mechanism of sudden death in CCC is arrhythmogenic. Sustained VT with subsequent ventricular fibrillation (VF) is responsible for the vast majority of lethal events[39]. In this sense, the structural abnormalities of CCC - with inflammation, cell death, and reactive and reparative fibrosis - constitute the ideal anatomical substrate because they promote unidirectional blocks and slow conduction areas conducive to triggering electrical reentry. The triggers that affect this anatomical substrate, the PVC, also invariably present, complete the essential elements for the installation of ventricular reentry tachyarrhythmia3[,18,20,22,27,34]. Thus, non-sustained VT can occur in approximately 40% of patients with CCC and regional changes in segmental mobility, and in virtually all patients with global LV systolic dysfunction and HF[40]. Sustained VT, with a more ominous prognosis, occurs spontaneously and can be reproduced in approximately 80-85% of patients during an electrophysiological study[17,18,41]. The complete atrioventricular block is another possible but less common cause of SCD in CCC, as a consequence of

necrotic degeneration and diffuse fibrosis predominantly in the atrioventricular region[19].

As mentioned above, SCD can also result from massive pulmonary thromboembolism or from a systemic embolic accident in vital organs, and, exceptionally, be a consequence of a ruptured left ventricular apical aneurysm.

Risk Stratification

As explained above, Rassi et al defined a score for risk stratification for mortality in patients with CCC, based on clinical variables and basic cardiological tests[11] (Tables 1 and 2).

The Rassi score, after its definition in an initial cohort, and after being externally validated in another independent cohort (Hospital Evandro Chagas, Rio de Janeiro), was also applied by other investigators in a third cohort analyzed retrospectively, of 149 patients, with similar results[42]. These investigators also proposed that the presence of VT on exercise testing or 24-hour Holter monitoring, LV ejection fraction (LVEF) less than 0.50, and QRS>150ms on high-resolution ECG (ECGHR) can identify patients with CCC and risk the highest mortality in five years. The absence of these factors or the presence of even one factor would characterize a low-risk group; the risk is considered intermediate when two factors occur, and high risk is characterized by the presence of all three factors. In light of these data, and also because the ECGHR is much less available and applicable, the Rassi score (already externally validated) is clearly superior for the purpose of patient selection in the context of this research project.

Antiarrhythmic therapeutic options

The therapeutic arsenal for fatal ventricular tachyarrhythmias and prevention of SCD includes antiarrhythmic drugs of various classes, catheter ablation or surgery, and implantable cardioverter-defibrillator (ICD)[43]. According to the I Latin American Guideline for the diagnosis and treatment of Chagas' cardiomyopathy[43], the use of amiodarone is a class I recommendation for the treatment of patients with CCC who present: a) symptomatic non-sustained VT and left ventricular dysfunction; b) Sustained VT, symptomatic or not, with or without left ventricular dysfunction, not treated with ICD; c) an implanted ICD, to reduce the frequency of appropriate shocks. Also according to this guideline, only the use of amiodarone in asymptomatic patients with CCC and non-sustained VT was indicated, even with left ventricular dysfunction. However, in another guideline, the assessment, and treatment of patients with cardiac arrhythmias recommends amiodarone as class IIa for patients with asymptomatic non-sustained VT and compromised ventricular function[44]. It is worth mentioning that, according to the results of recent longitudinal studies[11,45] the presence of non-sustained VT on Holter monitoring (especially if associated with impaired left ventricular function) is an important risk factor for mortality in CCC, regardless of the presence of symptoms[46]. The justification for the use of amiodarone in patients with non-sustained VT is supported by the following results: a) in the only two randomized studies that included chagasic patients, GESICA (n=516, 9.5% of chagasic patients)[47] and EPAMSA (n =127, 22% of chagasic patients)[48], the empirical administration of amiodarone in patients with HF was able to significantly reduce total mortality in both trials, after a mean follow-up of 13 and 12 months, respectively. Complex ventricular arrhythmia (paired PVC and/or episodes of non-sustained VT) was present in approximately 80% to 90% of patients and, in the GESICA study, the impact of amiodarone on reducing mortality was more pronounced in patients who had baseline non-sustained VT (from 57% to 44%) versus a death reduction from 34% to 28% in patients without non-sustained VT[47]; and b) in AMIOVIRT (n=103), the only randomized primary prevention study carried out in patients with dilated cardiomyopathy (non-chagasic) and who had non-sustained VT at

baseline, there was no difference in mortality between patients treated with the ICD and those treated with amiodarone[49].

In addition to possibly reducing mortality, amiodarone has extraordinary efficacy in suppressing ventricular ectopic beats, reduced incidence of major side effects, and pro-arrhythmia, particularly when administered in low doses[50]; in addition to its essential property as a Vaughan Williams class III drug, amiodarone also has a beta-adrenergic blocking effect (class II) demonstrated in experiments with animals stimulated by isoproterenol[51]; finally, it only very rarely affects cardiac inotropes in a clinically significant way, and can therefore be used, even in cases of severe impairment of left ventricular function[52,53].

On the other hand, the results of the SCD-HeFT study, which concluded that amiodarone is neutral in relation to placebo in primary prevention, should not be extrapolated to CCC. The SCD-HeFT (n=2521 – there was also a third arm that was randomized to the ICD) included patients with ischemic heart disease or idiopathic dilated cardiomyopathy (but not patients with CCC) whose LVEF was <35%. Non-sustained VT (present in 23% of patients on 24h- Holter monitoring) was not an inclusion criterion and most patients were on regular optimized use of beta-blockers[54]. Furthermore, among the numerous subgroup analyses performed, the one with the greatest relevance in the context, comparing amiodarone with placebo in patients with and without non-sustained VT, for unexplained reasons, was not performed by the authors of the study[54].

Regarding the use of the ICD in primary prevention in CCC, the I Latin American Guideline mentioned above, and recently published in the Arquivos Brasileiros de Cardiologia, concluded that "There is no scientific evidence to support the indication of ICD in the primary prevention of SCD, and therefore, at this time, there is no recommendation to suggest." Evolutionary results in a small series of 13 CCC patients treated for primary prevention with ICD were recently released, but obviously do not even offer evidence for any more authoritative conclusion about the efficacy or safety of this therapeutic intervention in CCC[55].

If the scenario of primary prevention of sudden death in CCC, whether pharmacologically or with ICD, is still completely devoid of scientific evidence to support its efficacy and safety, the context of secondary prevention is also filled

with profound uncertainties, as they have not yet been conducted any study with conclusive or definitive results.

Thus, the empirical use of amiodarone for the treatment of sustained VT, whose prevalence in CCC without HF is estimated at 2%, is associated with a recurrence rate of 40% and the probability of survival varies between 68% and 84% in three years of follow-up[56,57,58,59]. In a recently published series of 28 consecutive patients with sustained VT, all treated with amiodarone alone followed for approximately three years, the survival rate was 68%, with LV systolic dysfunction being the most significant predictor of death[60]. Annual mortality rates in patients with CCC and sustained VT treated with amiodarone alone ranged from 5.1% to 10.7%[56,60]. These data are quite favorable in comparison with those of other series (not simultaneous and not controlled), of patients not treated or medicated only with quinidine or procainamide, who have a survival probability of only 20%, considering the same follow-up time[3,14,34]. There is no unequivocal demonstration that the use of amiodarone, guided by an electrophysiological study[61], is superior to its empirical use, also because only about 29% of 71 patients with CCC and VT would be eligible for the treatment guided by the electrophysiologic study[57]. In other studies of association of amiodarone with class I antiarrhythmics or alternating with sotalol, the annual mortality rates were not lower than those of treatment with amiodarone alone, ranging from 8.8%[61] to 11.0%[62].

In summary, these results of treatment with amiodarone in patients with CCC are encouraging, although they cannot be directly compared to those described in patients with high-risk ischemic or dilated heart disease, in which the use of amiodarone was able to prevent SCD and reduce all causes mortality, according to the results of prospective studies[63,64] or meta-analyses[65,66].

Alternatively - when antiarrhythmic are not effective or are not tolerated - in small groups of selected patients with CCC, sustained VT can be corrected by endocardial or epicardial aneurysmectomy or catheter ablation surgery, appearing to reduce the likelihood of SCD[43,67]. However, such results are not applied, for obvious reasons, to most patients with CCC.

Regarding the use of ICDs for secondary prevention of SCD in CCC, scientific evidence is restricted to reports of small series or records of patients who received the device[68,69,70,71,72]. From these series, Martinelli-Filho et al and Muratore et al observed that appropriate ICD therapies (triggers) in chagasic patients are more frequent than in non-chagasic patients[68,69,70]. In addition, in the largest series of cases published[71] the results in 90 consecutive patients treated with ICD revealed a clearly unfavorable evolution compared to those obtained in other series of Chagas patients who were not treated with ICD, as indicated in an editorial referring to that work[73]. These surprising results occurred despite the fact that in this series of 90 patients, LV systolic function before ICD implantation was relatively well preserved. Also, as predictable based on the literature data on other heart diseases, it was found that the frequency of firings was associated with progression of ventricular dysfunction and death from refractory HF[71]. Therefore, the analysis of these studies suggests that the evolution of chagasic patients with ICD may not be as favorable as that observed in other heart diseases and that there are still many unclear points regarding the use of this therapeutic method in CCC, even in terms of secondary prevention[73].

Returning to the context of primary prevention of SCD, the criteria for indicating ICD in patients with CCC[74] are extrapolated from international studies, which predominantly included ischemic patients with severe ventricular dysfunction. Therefore, there is no report of minimally acceptable scientific evidence to endorse the systematic use of ICDs to primarily prevent SCD in CCC. Indeed, there are many pathogenic and pathophysiological peculiarities intrinsic to CCC, which make any direct comparison with the results in the literature in other cardiomyopathies difficult[6]. The most striking peculiarity is that many patients with CCC, even with preserved global ventricular function, already have a substrate for potentially lethal arrhythmias, as noted above and confirmed in numerous studies[20,34,39,40,41,75].

Despite these concepts and considerations, and even without an indisputable scientific basis, there is a relatively widespread perception that it would be difficult to justify carrying out a large-scale randomized study for the secondary

prevention of SCD in patients with CCC due to the prediction of obstacles of an ethical nature against a therapeutic alternative to the ICD[76].

It is currently estimated that no more than 1,000 devices are implanted in Latin America each year in patients with CCC. The recently published Latin American CDI Registry revealed that only 9% of cases are implanted for primary prevention of SCD and no evolutionary results were presented[72]. Among other factors responsible for these limitations, the finding that even for secondary prevention, the classic criteria of guidelines compiled for other clinical contexts do not apply to many patients with CCC, as they have potentially fatal arrhythmias in the presence of global LV function preserved[20,34,39,40,41,77].

In summary, there is still no scientific evidence regarding the role of the ICD in the primary prevention of SCD in CCC. In this sense, it is quite plausible and convenient to carry out a large-scale randomized controlled trial in this context.

Summary and Hypothesis

Chagas disease is an endemic problem in Latin America, where there are millions of people chronically infected with T. cruzi, and it has also recently assumed clinical and epidemiological relevance in several other countries due to migratory and globalizing social factors. CCC occurs in 30-50% of infected individuals, causing considerable morbidity/mortality rates. SCD is the main form of death in patients with CCC. While ICD implantation and amiodarone treatment have been advocated and performed empirically for secondary prevention in patients with CCC, there is no consistent scientific evidence on the role of these forms of treatment for the primary prevention of SCD in patients with CCC and high mortality risk[78].

The primary hypothesis of this study is that ICD implantation is more effective in the primary prevention of death in CCC than drug treatment with amiodarone in patients with documented non-sustained VT.

It should be noted that the risk of death will be assessed using the Rassi score, validated based on non-invasive variables, which, depending on the results of this study, may guide the indication of ICD in CCC.

Objectives

Primary Objective

- To compare the efficacy of ICD implant and amiodarone treatment in the primary prevention of all-cause death in patients with CCC and non-sustained VT.

Secondary Objectives

- To validate the use of the mortality risk stratification score for patients with CCC (Rassi score), as a criterion for indicating an ICD for primary prevention of SCD.
- To identify clinical and functional variables of worse prognosis in CCC according to the randomization arm.
- Record the worsening of HF, including the need for hospitalization, the evolution of the functional class according to the New York Heart Association classification, and the indication of cardiac resynchronization therapy according to the randomization arm.
- Record the occurrence of bradyarrhythmia requiring treatment with artificial pacemaker stimulation according to the randomization arm.

Outcome variables

Primary Endpoint

- Mortality from all causes;

Secondary Endpoints

- Cardiac mortality;
- Sudden cardiac death;
- Hospitalization due to heart failure worsening;

- Need of antibradycardia stimulation in the ICD arm and need for pacemaker implantation in the amiodarone treatment arm.

Methods

A prospective, Brazilian, open-label, multicenter study comparing outcomes in two parallel arms regarding treatment, with randomized allocation to each group, with an estimated inclusion period of 3 years and a minimum follow-up of 3 years after inclusion.

Population Sample

Approximately 1,100 patients with CCC will be recruited, stratified by the Rassi score[11], who meet the following inclusion and exclusion criteria:

Inclusion

- Signing an informed consent (ICF) before randomization and any study procedure;
- Both genders, age >18 years and <75 years;
- Recent positive serology (last 2 years) and documented for Chagas disease, in at least two different tests (indirect hemagglutination, indirect immunofluorescence, or ELISA);
- Presence of at least 10 points in the Rassi score, with mandatory presence of at least 1 episode of non-sustained VT, defined as >3 successive beats and duration <30 seconds, with HR >100 bpm.

Exclusion

- Participation in another study, currently or completed <1 year ago, with the exception of a totally unrelated observational study;

- Another concomitant cardiovascular disease, including uncontrolled diabetes mellitus (systemic arterial hypertension without end-organ involvement permitted);
- Renal (serum creatinine >1.5 mg/dL or eGFR <30mL/min/1.73m2) or liver dysfunction, with a diagnosis of cirrhosis or portal hypertension or elevation of serum enzymes (AST or ALT) >3x the upper limit of normal;
- Moderate or severe chronic obstructive pulmonary disease;
- Peripheral polyneuropathy;
- Hyperthyroidism;
- Current or non-abandoned drinking for > 2 years;
- With a diagnosis of psychopathy or psychosis or addiction to illicit drugs;
- Life expectancy <1 year, due to disease itself or co-morbidities (including NYHA class IV CHF);
- Pregnancy or breastfeeding;
- Potential to become pregnant during the study (non-menopausal patients, and who have not undergone a radical and safe contraceptive process);
- Other contraindications for the use of amiodarone: previous intolerance to the drug;
- Formal indication for use of amiodarone or ICD;
- Use of amiodarone in the last 6 months, unless started <2 weeks ago and whose loading dose was <10g, and the maintenance dose was ≤200mg/day;
- Current use of other drugs with contraindication to the concomitant use of amiodarone under a therapeutic regimen that may be associated with a proarrhythmic effect;
- Withdrawal from this study, previously.

Study Flowchart and Steps

During the selection process, and for the purpose of applying the inclusion and exclusion criteria, the following complimentary assessments will always be carried out: complete anamnesis, including specific determination of the **HF functional class of HF (NYHA)** and current use of medications, cardiovascular physical

examination, 12-lead EKG at rest, chest X-ray, two-dimensional transthoracic echocardiogram and 24-h Holter monitoring (Figure 3). This initial clinical evaluation prior to randomization should include biochemical analysis of the blood for complete blood count, magnesium, potassium, creatinine, urea, TGP, TGO, TSH, and free T4.

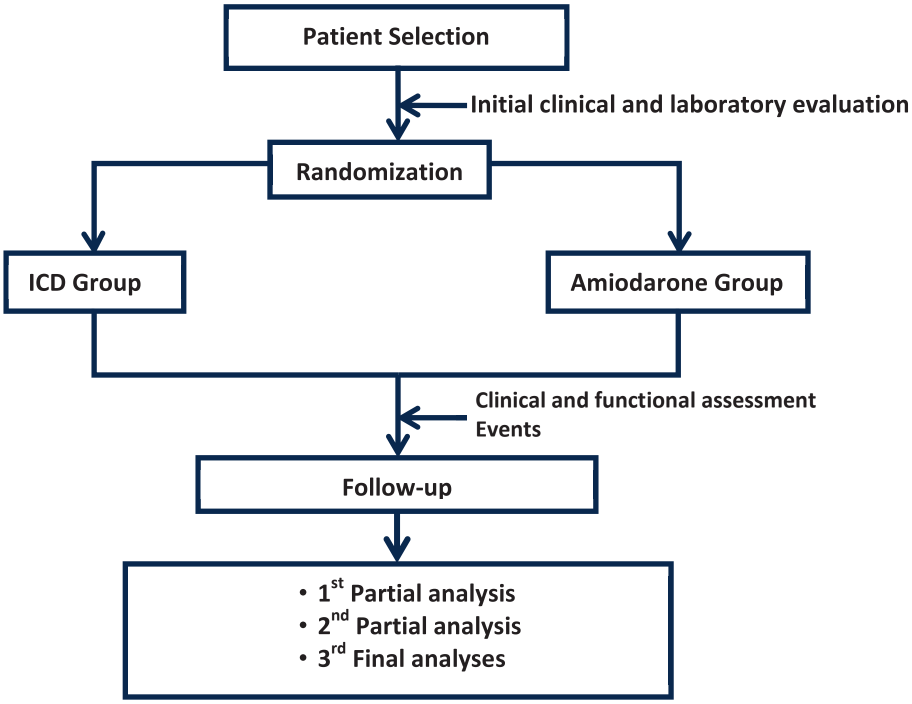

Figure 3. Study flowchart

Allocation List Randomization and confidentiality

Patients selected based on inclusion and exclusion criteria will be randomized to ICD implantation or treatment with amiodarone, at a 1:1 ratio.

The random allocation process will be carried out by an automated and central system (type IWRS) for all participating centers and confidentiality will be guaranteed by this same system. In the event of lack of financial resources, the list will be generated by the "random" function of MS Excel and confidentiality will be guaranteed by means of envelopes, opaque, sealed, and sequentially numbered.

ICD Group

ICD implantation must be performed according to the institutional protocol of each participating center; the device should preferably be unicameral and the programming should prioritize the proper rhythm, avoiding ventricular pacing.

After the initial visit, follow-up visits will be scheduled for 10 days, 1, 4, 8, 12, 16, 20, 24, 28, 32, and 36 months; variation of a maximum of two weeks will be tolerated for scheduled visits (more or less), otherwise, they will be considered unscheduled visits.

St. Jude Medical Brasil will provide technical support for the evaluation of implanted devices.

In this group, the use of amiodarone should be avoided, except in cases of multiple ICD shocks, refractory to beta-blockers (including Sotalol hydrochloride, and/or catheter ablation.

Amiodarone Group

Patients randomized to this group will use amiodarone hydrochloride (once a day), according to the scheme below:

- The initial dose of 600 mg/day, administered orally for 10 days, in an outpatient setting, for impregnation;
- After the impregnation period, an oral dose between 200 and 400mg/day should be maintained until the end of the study. It will be up to each investigator to choose the ideal maintenance dose, which may be guided by the therapeutic response to 24h Holter monitoring, HR at rest, side effects, QTc interval prolongation, etc. Dosage adjustments will be allowed throughout the study period as long as the maintenance dose is between 200 and 400mg/day. If the patient does not tolerate a minimum dose of 200mg/day, amiodarone should be permanently discontinued and the treatment considered interrupted.

Clinical Follow-up

All selected patients must be followed up for at least 3 years.

In all scheduled visits, the following procedures will be carried out:

- HF NYHA Functional Class Assessment;
- Record of changes to medications in use;
- Counting of amiodarone tablets and recording the maintenance dose used;
- Electronic ICD assessment.

Additional procedures will be carried out on visits scheduled for 12, 24, and 36 months:

- 12-lead electrocardiogram, at rest;
- Transthoracic 2D-echocardiogram;
- Biochemical Exams.

24-h Holter monitoring should be performed at 10 days, 4 months, and 12 months.

Unscheduled visits

- Any unplanned contact between the patient and the study investigator will be considered an unscheduled visit; the data collected and procedures performed in these visits will follow the routine of the scheduled visits.

Deaths Classification

- The circumstances of the deaths will be reviewed for characterization of cardiac or non-cardiac causes and the definitions of Hinkle and Thaler should be used to assess the possible suspected mechanisms of the cause of death. The adjudication of deaths and other outcomes will be the responsibility of a Specific Committee of three members, independent from the Data Security and Monitoring Committee.

Heart failure hospitalization

- Hospital admissions for worsening HF will be considered the ones including visits to the emergency department for the treatment of HF (International

Classification of Diseases, 10th revision – ICD-10 = I50) or acute pulmonary edema (ICD-10 = J81) that require increased medication and include administration of intravenous positive vasoactive or inotropic drugs.

Statistical Plan and Sample Size Calculation

Considering a mortality rate of 30% in the amiodarone-treated group after a mean follow-up time of 4.5 years and a relative risk reduction of at least 30% with the ICD (the mortality rate falling to, at least 21%), and adopting an error of $\alpha=0.05$, power of 90% and follow-up losses of up to 10%, the total sample size is approximately 1,100 patients (that is, 550 in each arm). The CHAGASICS study will be event-driven, so it will continue until at least 256 events are observed. Thus, if this number of events is reached with less than 1,100 patients, the inclusion of patients will be terminated. The advantage of using the event-driven strategy is to guarantee to the study satisfactory statistical power and avoid recruiting an inadequate number of patients.

This sample size calculation also contemplates the execution of two interim analyzes that will be carried out by an independent committee when one-third and two-thirds of the events have occurred. In this sense, the study may also be interrupted (for safety, benefit, or futility) as recommended by the Independent Data Security and Monitoring Committee. The method that will be used by this Committee is that of O'Brien & Fleming, which considers different values of α for each interim analysis, adopting extremely conservative values at the beginning of the study, when the sample is small, and values closer to the pre-established when it is close to ending the study and will be used to guide the premature interruption of the study[79,80].

Statistical methods

The groups will be compared under the principle of intention-to-treat analysis. If there is a “crossover” above 20% (in any direction), a sensitivity analysis will also be performed using the principle of “on treatment” analysis.

Data will be expressed as mean and standard deviation or as a proportion.

Categorical variables will be analyzed using the chi-square test. Continuous data will be compared by the t-test (normal distribution) or non-parametric Mann-Whitney test (non-normal distribution).

Cumulative events will be calculated using the Kaplan-Meier method and the log-rank test will be used to compare groups.

Hazard Ratio (Hazard Ratio) with 95% confidence interval will be calculated using Cox regression analysis; the same methodology will be used for the analysis of subgroups.

The statistical tests will be two-tailed and the value of $P<0.05$ will be considered significant.

To organize the data and results, a relational database with SQL structure will be used and the statistical analyzes will be performed by SPSS version 16.0 for Windows.

Planned subgroup analysis

Subgroup analysis should include gender, age ≥ or <60 years, presence or absence of atrial fibrillation, functional class (NYHA) I and II versus III and IV as well as Rassi score.

Planned sub-study

Unrelated observational sub-studies are allowed to preserve the therapeutic approach of the CHAGASICS study. The related sub-study to be carried out will be the cost-effectiveness sub-study.

Special Considerations

The systematization of special clinical care procedures in relation to events during follow-up must comply with the criteria below:

1) **Amiodarone intolerance:** after discontinuation of the drug, the introduction or increase of the beta-blocker dose should be considered;
2) **Occurrence of sustained VT/VF:** consideration should be given to increasing the dose of amiodarone and/or beta-blocker.

   At the physician's discretion, catheter ablation may be performed.

   In the case of the amiodarone group, after catheter ablation, the investigator may choose to perform ICD implantation (crossover), which should be delayed as far as possible.

   Specifically for the ICD arm, in case of an arrhythmic storm or very frequent and symptomatic ventricular arrhythmia, the first complementary therapeutic measure should be catheter ablation and, in case of failure, administration of amiodarone (crossover) and/or adjustment of the beta-blocker dose;
3) **Severe sinus bradycardia or AV block** will indicate permanent pacemaker implantation; the patient should not be excluded from the study;
4) **Drug association:** angiotensin-converting enzyme inhibitors, angiotensin receptor blockers, beta-blockers, spironolactone, antiplatelet drugs, etc. may be administered at the discretion of the attending physician;
5) **The ICD programming must comply with the following criteria:** pacemaker VVI mode with pacing rate <40; monitoring zone (therapy off) of ventricular tachycardia starting at 120 bpm; anti-tachycardia function for shock therapy adjusted for the cut-off rate of 180 bpm and maximum energy. In the event of sustained VT, ATP programming must be activated, using a burst of 8 pulses programmed between 80 and 90% of the RR cycle of the VT and repetitions at physician’s discretion and back-up shock therapy.

Criteria for Discontinuation of Amiodarone

Definitive Interruption

1. Pro-arrhythmia
    a. Increased density of PVC on 24h-Holter monitoring

Basal density (Average of PVC/h)	Increase compatible with proarrhythmia (Average of PVC/h)
10-100	7x
100-300	4x
300-1000	3x
>1000	2x

    b. *non-sustained TV episodes increasing*

Basal density (non-sustained VT/24h)	Increase compatible with proarrhythmia
<5	≥ 50 episodes/24h
≥ 5	>10x

    c. *Torsades de pointes*

2. Liver changes:
    a. Increased bilirubin, TGO, TGP, or alkaline phosphatase by at least 3x the upper limit of normal
    b. Increased bilirubin, TGO, TGP, or alkaline phosphatase between 2-3x for 2 or more months
3. Induced thyroid disorders
    a. Symptomatic hyperthyroidism
    b. Hypothyroidism
4. Ocular adverse effects

   a. Corneal microdeposits of great intensity with blurring of vision or perception of a colored halo around a light source look at by the patient.
5. QTc interval prolongation (>500ms) and/or QTc interval dispersion (>80ms).

Discontinuation at Investigator/Patient discretion or dose reduction

1. Neurological disorders:
   a. Ataxia and hand tremor
2. Ataxia and hand tremor:
   a. Ataxia and hand tremor
   b. Skin hyperchromia
3. Digestive disorders:
   a. Nausea, vomiting and constipation

Notes on interruption

1. Increased TSH (asymptomatic): close observation
2. Symptomatic hypothyroidism: initiate l-thyroxine, continue treatment at investigator's discretion
3. Appearance of radiological changes (interstitial and/or diffuse and bilateral alveolar infiltrate) not explained by HF or pulmonary infection: interrupt treatment and perform additional tests such as pulmonary gallium scintigraphy, bronchoscopy + lung biopsy, etc.; corticosteroid can be used; treatment can be restarted depending on the results.

Adverse Events

For the purposes of this study, adverse events will be considered any unfavorable events that occur after signing the informed consent.
Pre-planned procedures and pre-existing conditions documented at recruitment should be recorded as medical history and should not be considered adverse

events unless a worsening of a condition already in place at that time has occurred.

Adverse events will be classified according to severity, causality and outcome, and relationship to medication or studied product. It will be considered:

1. For patients in the amiodarone group - pro-arrhythmia, thyroid disorders, hepatotoxicity, corneal or induced skin pigmentation, neuropathy disorders, digestive disorders, and pulmonary fibrosis[50,51,81,82];
2. For patients in the ICD group - infection at the implantation site of the pulse generator or lead, malfunctions related to the pulse generator or leads, including inappropriate therapies[83,84].

Serious Adverse Events

They must be reported, within a maximum period of 24-h after event documentation, to the Study Coordinating Center and to the Research Ethics Committee of the current center and will be considered:

1. Deaths from any cause - If the death occurs in a patient of the ICD group, a complete evaluation of the device must be performed, accompanied by a printed record of the electronic data.
2. Hospitalizations or prolongation of hospital stay - it will be considered any hospitalization for a period longer than 24h and any situation that leads to a prolongation of the patient's hospital stay, such as sepsis, worsening HF, ICD implant complications, etc.

Protocol Deviations

Cases of non-compliance with the study rules will be considered protocol deviations. These must be documented and reported, within 30 days after they occur to the Coordinating Center, which will determine the procedure to be performed.

The Research Ethical Committee of each Institution should also be communicated about these deviations through the periodic monitoring reports of the study.

Risks and Benefits

Risks to the Patient

Patient risks include known complications and adverse events related to ICD implant procedures and to the use of amiodarone[55-58].

Patient Benefits

The risk stratification applied to recruited patients and the definition of the necessary treatment will result in a beneficial contribution inherent to the subject participating in the research.

Regardless of the type of treatment to which the patient has been randomized, everyone will be closely monitored to ensure immediate clinical interventions that tend to correct any disturbances linked to the evolution of the underlying disease or other clinical problems that appear.

The study drug (amiodarone) and the ICD will be given to patients free of charge. Each investigator and research center involved in the study must also ensure that other drugs indicated to patients are made available at the lowest possible social cost.

There will be no payment to the patient for participating in the study.

Study Organization

The Coordinating Center of the study will be the Instituto do Coração (InCor) of the Hospital das Clínicas of the Faculty of Medicine of the University of São Paulo and the coordinator and principal clinical investigator of the study will be Prof. Martino Martinelli Filho.

The study's organizational chart includes the composition of Steering Committee; Data Security and Monitoring Committee; Editorial Committee; Research Executive Committee; Event Adjudication Committee, Research Centers, Principal, and Secondary Investigators, and Research Coordinators at each Center.

The Steering Committee will have the function of reviewing and monitoring all phases of the study on a regular basis. Based on this close monitoring, the Committee may determine the continuation or interruption of the study at any time, considering ethical or safety circumstances. This Commission will have the services of an Executive Secretary who will be its liaison with the other instances of project administration. This committee is chaired by Prof. Martino Martinelli Filho and the other members are: Prof. José Antonio Marin-Neto, Prof. Angelo Amato Vincenzo de Paola; Prof. Maurício Ibrahim Scanavacca, Prof. Anis Rassi Jr. and the Biomedical Engineer Sérgio Freitas de Siqueira.

The Editorial Committee will be responsible for defining the editorial standards in addition to being responsible for the material for publication and presentation at scientific conferences. This committee will be chaired by Prof. Martino Martinelli Filho who will have, among others, the participation of Prof. Roberto Costa as active members. According to the need and potential for collaboration, new members will be invited by the president, always following the authorship criteria established by the International Committee of Medical Journal Editors (ICMJE), whose criteria for authorship credit is based exclusively on substantial contributions: (i) the conception and design, or the acquisition of data, or the analysis and interpretation of data; (ii) the writing of the article or its substantial revision, regarding relevant intellectual content; and (iii) approval of the final version to be published.

The Data Safety and Independent Monitoring Committee shall conduct periodic analysis of results and adverse events, recommending changes and eventual interruption of the study to the Steering Committee. This Committee will have the support of a biostatistics professional. This committee will be appointed by Prof. Martino Martinelli Filho at an opportune moment.

The Executive Committee will be responsible for managing and monitoring the study, ensuring the quality of the information. It should be responsible for preparing relevant reports to regulatory instances.

The Event Adjudication Committee will work with three members designated by the Study Steering Committee, with data provided by the Executive Committee.

The Research Centers will be responsible for enforcing the phases of the study in accordance with good clinical practices, providing quality data and reports of adverse events.

Ethical and Regulatory Basis and Data Quality Control

This study was designed and must be conducted in accordance with the principles established in the most current version of the Declaration of Helsinki[59] and in accordance with relevant national regulations, following all the requirements determined by the law and specific regulations on the matter, especially Resolution 196/96 from National Health Council. The precepts of good clinical practice will be followed in conducting the study according to Document of the Americas, Pan American Health Organization, IV Pan American Conference for Harmonization of Pharmaceutical Regulations Dominican Republic, March 2-4, 2005.

Approval of the study and the ICF by the Ethics Committee of each Center will be required prior to the beginning of the study and patient recruitment. In accordance with current regulations, National Ethics Committee (CONEP) will be duly instructed by the Coordinator Center.

Timeline

The period required to obtain study approvals at the various centers is estimated at a maximum of one year. The enrollment of patients will remain open until the predicted number is reached and should continue for an estimated period of three consecutive years. The follow-up period will be three years for each patient recruited. Thus, the duration of the study should vary between four and seven years, with a minimum follow-up of three and a maximum initially planned of six years for the first patients who enter the study, depending on the results of the interim analyses.

Budget

The estimated cost of the project is related to the tests required for the clinical follow-up of the patients enrolled, according to the table below:

Amount	Test	Per unit	Per patient
12	Clinical and Electronic assessment	125.00	1.500.00
4	12-lead electrocardiogram	21.84	87.36
4	24-h Holter monitoring (3 digital channels)	136.60	546.40
4	Echocardiogram (two-dimensional color flow)	207.40	829.60
4	Complete blood count	8.78	35.12
1	2-view chest radiography	25.64	25.64
1	Creatinine	3.95	3.95
1	AST	7.49	7.49
1	ALT	7.49	7.49
1	Magnesium	3.95	3.95
1	Potassium	3.95	3.95
1	Thyroid function tests (TSH, T4, T3)	61.31	61.31
1	Serologic test for Chagas (EIE)	18.46	18.46
1	Serologic test for Chagas (HA)	9.35	9.35
	**Total sum per patient**		**3,140.07**

Costs for hiring a statistician is estimated at R$ 8000.00, of which two thousand will be spent in each interim analysis; two thousand will be set aside for occasional extra analyses.

SPONSORS: For these basic costs, financial support was obtained from the company St. Jude Medical Brazil.

Expenses for researchers meetings and for hiring IT and monitoring services should be obtained from institutions fostering research such as FAPESP, DECIT and CNPq.

Bibliographic references

1 Chagas C. Nouvelle espèce de trypanosomiase humaine. Bull Soc Path Exotique. 1909;6:304-7.

2 Dias JCP. História natural da doença de Chagas. Arq Bras Cardiol. 1995;65(4):359-66.

3 Rassi A J r, Ras s i A, Mar in-Neto JA. Chagas di sease. Lancet. 2010;375(9723):1388-402.

4 Dias JC. The indeterminate form of human chronic Chagas' disease: a clinical epidemiological review. Rev Soc Bras Med Trop. 1989;22(3):147- 56.

5 Barretto AC, Ianni BM. The undetermined form of Chagas' heart disease: concept and forensic implications. Sao Paulo Med J 1995.

6 Marin-Neto JA, Cunha-Neto E, Maciel BC, Simões MV. Pathogenesis of chronic Chagas heart disease. Circulation.2007;115:1109-23.

7 Schmunis GA. Epidemiology of Chagas disease in nonendemic countries: the role of international migration. Mem Inst Oswaldo Cruz. 2007;102 Suppl 1:75-85.

8 Dias JC, Prata A, Correia D. Problems and perspectives for Chagas disease control: in search of a realistic analysis. Rev Soc Bras Med Trop. 2008;41:193-6.

9 Moncayo A, Silveira AC. Current epidemiological trends for Chagas disease in Latin America and future challenges in epidemiology, surveillance and health policy. Mem Inst Oswaldo Cruz. 2009;104(Supl 1):17-30.

10 RassiJr A, Dias JCP, Marin-Neto J A, Rassi A. Challenges and opportunities for primary, secondary, and tertiary prevention of Chagas' disease Heart 2009;95:524–534.

11 RassiJr.A, Rassi A, Little WC, Xavier SS, Rassi SG, Rassi AG, Rassi GG, Hasslocher-Moreno A, Sousa AS, Scanavacca MI. Development and Validation of a Risk Score for Predicting Death in Chagas' Heart Disease. N Engl J Med 2006;355:799-808.

12 Dias JCP, Kloetzel K. The prognostic value of the electrocardiographic features of chronic Chagas'disease. Rev Inst Med Trop São Paulo 1968; 10: 158-62.

13 Espinosa R, Carrasco HA, Belandria F, et al. Life expectancy analysis in patients with Chagas'disease: prognosis after one decade (1973-1983). Int J Cardiol 1985; 8:45-56.

14 Rassi A. Curva atuarial da taquicardia ventricular sustentada na cardiopatia chagásica crônica. In: Anais do IV Simpósio Brasileiro de Arritmias Cardíacas. Recife 1987: 129.

15 Santana OO. Arritmia ventricular e evolução clínica de pacientes na fase crônica da doença de Chagas (Dissertação de Mestrado). Salvador: Universidade Federal da Bahia, 1987: 50p.

16 Carrasco HA. Factores pronosticos en la evolucion de la cardiopatiachagasicacronica. Rev Fed Arg Cardiol 1988; 17: 247-50.

17 Rassi SG, Rassi Jr A, Rassi AG, Lima AMC, Jatene JA, Rassi A. Avaliação da síncope e da pré-sincope na cardiopatia chagásica crônica através da estimulação elétrica programada. In: Anais do 2º Congresso da Sociedade Latino-Americana de Estimulação Cardíaca. Porto Alegre, 1989: 36.

18 Rassi SG, Rassi Jr A, Jatene JA, Lima AMC, Ghannam VM, Rassi A. Significado clínico da indução de fibrilação ventricular, flutter ventricular e taquicardia ventricular polimórfica sustentados ao estudo eletrofisiológico. Arq Bras Cardiol 1991; 57(supl C): C2.

19 Rassi A, Rassi Jr A, Faria GHDC, et al. História natural do bloqueio atrioventricular total de etiologia chagásica. Arq Bras Cardiol 1992; 59(supl II): 191.

20 Rassi Jr A, Rassi AG, Rassi SG, Rassi Jr L, Rassi A. Relação entre sintomas, disfunção ventricular e arritmia ventricular na cardiopatia chagásica crônica. Arq Bras Cardiol 1992; 59(supl II): 182.

21 Carrasco HA, Parada H, Guerrero L, Duque M, Durán D, Molina C. Prognostic implications of clinical, electrocardiographic and hemodynamic findings in chronic Chagas' disease. Int J Cardiol 1994; 43: 27-38.

22 Scanavacca M, Sosa E. Estudo eletrofisiológico na cardiopatia chagásica crônica. Rev Soc Cardiol Estado São Paulo 1994: 2:168-76.

23 Moraes AP, Moffa PJ, Sosa EA, et al. Eletrocardiograma de alta resolução na cardiopatia chagásica crônica. Rev Soc Cardiol Estado São Paulo 1994; 4: 177-82.

24 MartinelliFilho M, Sosa E, Nishioka S, Scanavacca M, Bellotti G, Pileggi F. Clinical and electrophysiologic features of syncope in chronic chagasic heart disease. J Cardiovasc Electrophysiol 1994; 5: 563-70.

25 Mady C, Cardoso RHA, Pereira-Barretto AC, Luz PL, Bellotti G, Pileggi F. Survival and predictors of survival in patients with congestive heart failure due to Chagas' cardiomyopathy. Circulation 1994; 90: 3098-102.

26 Bestetti RB, Dalbo CMR, Freitas OC, Teno LAC, Castilho OT, Oliveira JSM. Noninvasive predictors of mortality for patients with Chagas' heart disease: a multivariate stepwise logistic regression study. Cardiology 1994; 84:261-7.

27 De Paola AAV, Gomes JA, Terzian AB, Miyamoto MH, Martinez EE. Ventricular tachycardia during exercise testing as a predictor of sudden death in patients with chronic chagasic cardiomyopathy and ventricular arrhythmias. Br Heart J 1995; 74:293-5.

28 Bestetti RB, Dalbo CM, Arruda CA, Correia Filho D, Freitas OC. Predictors of sudden cardiac death for patients with Chagas' disease: a hospital-derived cohort study. Cardiology 1996; 87:481-7.

29 Silva RMFL. Valor preditivo das variáveis clínicas e eletrofisiológicas nos pacientes com cardiopatia chagásica crônica e taquicardia ventricular não-sustentada Análise terapêutica (Tese). São Paulo: Escola Paulista de Medicina da Universidade Federal de São Paulo, 1997:147p.

30 Garzon SAC, Lorga AM, Jacob JLB, et al. Predictors of mortality in chronic Chagas heart disease long-term follow-up of 987 subjects for up to 22 years. J Am Coll Cardiol 1998; 31(suppl C):107C.

31 Rassi Jr A, Waktare JEP, Rassi SG, et al. Chagas heart disease: long term prognostic significance of nonsustained ventricular tachycardia and left ventricular dysfunction. PACE 1999; 22(Part II):862.

32 Carrasco HA, Guerrero L, Parada H, Molina C, Vegas E, Chuecos R. Ventricular arrhythmias and left ventricular myocardial function in chronic chagasic patients. Int J Cardiol 1990;28:35–41.

33 Lopes ER: Sudden death in patients with Chagas disease. Mem Inst Oswaldo Cruz 1999;94(Suppl 1):321-324.

34 Rassi A Jr, Rassi SG, Rassi AG, et al. Sudden death in Chagas disease. Arq Bras Cardiol 2001;76:75–96.

35 Bestetti RB. Stroke in a hospital-derived cohort of patients with chronic Chagas' disease. Acta Cardiol 2000;55:33–8.

36 Carod-Artal FJ, Vargas AP, Horan TA, Nunes LG. Chagasic cardiomyopathy is independently associated with ischemic stroke in Chagas' disease. Stroke 2005;36:965–70.

37 Sousa AS, Xavier SS, Freitas GR, Hasslocher-Moreno A. Prevention strategies of cardioembolic ischemic stroke in Chagas' disease. Arq Bras Cardiol. 2008 Nov;91(5):306-10.

38 Oliveira JSM, Barbieri–Neto J. Cardiopatia Chagásica. “Aneurisma da Ponta” roto. Arq Bras Cardiol 1970;23:335–8.

39 Mendoza I, Moleiro F, Marques. Morte súbita na doença de Chagas. Arq Bras Cardiol 1992;59:3–4.

40 Rassi Jr A, Rassi AG, Rassi SG, Rassi Jr L, Rassi A. Arritmias ventriculares na doença de Chagas. Particularidades diagnósticas,prognósticas e terapêuticas. Arq Bras Cardiol 1995;65:377–87.

41 Mendoza I, Camardo J, Moleiro F, Castellanos A, Medina V, Gomez J, Acquatella H, Casal H, Tortoledo F, Puigbo J. Sustained ventricular tachycardia in chronic chagasic myocarditis: electrophysiologic and pharmacologic characteristics. Am J Cardiol. 1986 Feb 15;57(6):423-7.

42 Rocha MO, Ribeiro AL. A risk score for predicting death in Chagas' heart disease. N Engl J Med. 2006; 355(23):2488-9; author reply 2490-1.

43 Andrade J.A., Marin-Neto J.A., Paola A.A.V., Vilas-Boas F., Oliveira G.M.M., Bacal F., Bocchi E.A, Almeida D.R., Fragata Filho A.A., Moreira M.C.V., Xavier S.S., Oliveira Junior W. A., Dias J.C.P. et al. Sociedade Brasileira de Cardiologia. I Diretriz Latino Americana para o Diagnóstico e Tratamento da Cardiopatia Chagásica. Arq Bras Cardiol 2011; 97(2 supl.3):1-48.

44 Scanavacca MI, Brito FB, Maia I, Hachul D, Gizzi J, Lorga A, et al. Diretrizes para Avaliação e Tratamento de Pacientes com Arritmias Cardíacas. Arq Bras Cardiol. 2002;79 (supl.V):1-50.

45 Ribeiro ALP, Cavalcanti PS, Lombardi F, Nunes MCP, Barros MVL, Rocha MOC. Prognostic Value of Signal-Averaged Electrocardiogram in Chagas Disease. J Cardiovasc Electrophysiol. 2008;19:502-509.

46 Rassi A Jr, Rassi A, Rassi SG.Predictors of mortality in chronic Chagas disease: a systematic review of observational studies. Circulation. 2007 Mar 6;115(9):1101-8. Review.

47 Doval HC, Nul DR, Grancelli HO, Perrone SV, Bortman GR, Curiel R. Randomised trial of low-dose amiodarone in severe congestive heart failure. Grupo de Estudio de la Sobrevida en la Insuficiencia Cardiaca en Argentina (GESICA). Lancet. 1994 Aug 20;344(8921):493-8.

48 Garguichevich JJ, Ramos JL, Gambarte A, Gentile A, Hauad S, Scapin O, Sirena J, Tibaldi M, Toplikar J.Effect of amiodarone therapy on mortality in patients with left ventricular dysfunction and asymptomatic complex ventricular arrhythmias: Argentine Pilot Study of Sudden Death and Amiodarone (EPAMSA). Am Heart J. 1995 Sep;130(3 Pt 1):494-500.

49 Strickberger SA, Hummel JD, Bartlett TG, Frumin HI, Schuger CD, Beau SL, Bitar C, Morady F; AMIOVIRT Investigators. Amiodarone versus implantable cardioverter-defibrillator: randomized trial in patients with nonischemic dilated cardiomyopathy and asymptomatic nonsustained ventricular tachycardia--AMIOVIRT. J Am Coll Cardiol. 2003 May 21;41(10):1707-12.

50 Rosenbaum MB et al. Clinical efficacy of amiodarone as an antiarrhythmic agent. Am J Cardiol. 1976; 38:934.

51 Harris L, Chatelain P, Nokin P, Roncucci R. Pharmacology of amiodarone. In Amiodarone. Pharmacology, pharmacokinetics, toxicology, clinical effects. Editors: Harris L & Roncucci R. Médecine et Sciences Internationales, Paris, 1986, pg 23-24.

52 Trobaugh GV et al. Amiodarone effects on ventricular function. Circulation 1983; 68:1117-

53 Pfisterer M et al. Amiodarone depresses cardiac function acutely but not chronically. Circulation 1983; 68:1122-

54 Bardy GH, Lee KL, Mark DB, Poole JE, Packer DL, Boineau R, Domanski M, Troutman C, Anderson J, Johnson G, McNulty SE, Clapp-Channing N, Davidson-Ray LD, Fraulo ES, Fishbein DP, Luceri RM, Ip JH; Sudden Cardiac Death in Heart Failure Trial (SCD-HeFT) Investigators. Amiodarone or an implantable

cardioverter-defibrillator for congestive heart failure. N Engl J Med. 2005 Jan 20;352(3):225-37. Erratum in: N Engl J Med. 2005 May 19;352(20):2146.

55 Cardinalli-Neto A, Nakazone MA, Grassi LV, Tavares BG, Bestetti RB. Implantable Cardioverter-Defibrillator therapy for primary prevention of sudden cardiac death in patients with severe Chagas cardiomyopathy. Int J Cardiol. 2011 Jul 1;150(1):94-5.

56 Scanavacca MI, Sosa EA, Lee JH, Bellotti G, Pileggi F. Terapêutica empírica com amiodarona em portadores de miocardiopatia chagásica crônica e taquicardia ventricular sustentada. Arq Bras Cardiol1990;54:367–71.

57 Giniger AG, Retyk EO, Laiño RA, Sananes EG, Lapuente AR. Ventricular tachycardia in Chagas' disease. Am J Cardiol 1992;70:459–62.

58 Sosa E, Scanavacca M, DÁvila A, et al. Endocardial and epicardial ablation guided by nonsurgical transthoracic epicardial mapping to treat recurrent ventricular tachycardia. J Am Coll Cardiol 1998;9:229–39.

59 Leite LR, Fenelon G, Paes AT, de Paola AAV. The impact of syncope during clinical presentation of sustained ventricular tachycardia on total and cardiac mortality in patients with chronic chagasic heart disease. Arq Bras Cardiol2001;77:446–52.

60 Sarabanda AV, Marin-Neto JA. Predictors of mortality in patients with Chagas' cardiomyopathy and ventricular tachycardia not treated with implantable cardioverter-defibrillators. Pacing Clin Electrophysiol. 2011 Jan;34(1):54-62.

61 Leite LR, Fenelon G, Simoes A Jr, Silva GG, Friedman PA, de Paola AA. Clinical usefulness of electrophysiologic testing in patients with ventricular tachycardia and chronic chagasic cardiomyopathy treated with amiodarone or sotalol. J Cardiovasc Electrophysiol. 2003 Jun;14(6):567-73.

62 Lorga Filho A. Influencia da presença de taquicardia ventricular sustentada na sobrevivência a longo prazo de pacientes chagásicos tratados clinicamente: um estudo caso-controle. São Paulo, 2002. 104p Doutorado Faculdade de Medicina da USP.

63 Burkart F, Pfisterer M, Kiowski W, Follath F, Buckhardt D. Effect of antiarrhythmic therapy on mortality in survivors of myocardial infarction with asymptomatic complex ventricular arrhythmias: Basel Antiarrhythmic Study of Infarct Survival (BASIS). J Am Coll Cardiol 1990; 16:1711-8.

64 Cairns JA, Connolly SJ, Roberts R, Gent M, for the Canadian Amiodarone Myocardial Infarction Arrhythmia Trial Investigators. Randomised trial of outcome after myocardial infarction in patients with frequent or repetitive ventricular premature depolarisations: CAMIAT. Lancet 1997; 349: 675-82.

65 Sim I, McDonald KM, Lavori PW, Norbutas CM, Hlatky MA. Quantitative overview of randomized trials of amiodarone to prevent sudden cardiac death. Circulation 1997; 96: 2823-9.

66 ATMA Investigators. Effect of prophylactic amiodarone on mortality after acute myocardial infarction and in congestive heart failure: meta-analysis of individual data from 6500 patients in randomised trials. Lancet 1997; 350:1417-24.

67 Sosa E, Scanavacca M, DÁvila A, et al. Endocardial and epicardial ablation guided by nonsurgical transthoracic epicardial mapping to treat recurrent ventricular tachycardia. J Am CollCardiol 1998;9:229–39.

68 Muratore C, Rabinovich R, Iglesias R, Gonzalez M, Darú V, Liprandi AS. Implantable cardioverter-defibrillators in patients with Chagas' disease: are they different from patients with coronary disease? Pacing Clin Electrophysiol 1997;20:194–7.

69 Rabinovich R, Muratore C, Iglesias R, et al. Time to first shock in implantable cardioverter defibrillator patients with Chagas cardiomyopathy. Pacing Clin Electrophysiol 1999;22:202–5.

70 Martinelli-Filho M, Siqueira SF, Moreira H, et al. Probability of occurrence of life-threatening ventricular arrhythmias in Chagas' disease versus non-Chagas' disease. Pacing Clin Electrophysiol 2000;23:1944–8.

71 Cardinalli-Neto A, Bestetti RB, Cordeiro JA, Rodrigues VC. Predictors of all-cause mortality for patients with chronic Chagas' heart disease receiving implantable cardioverter-defibrillator therapy. J Cardiovasc Electrophysiol 2007;18:1236–40.

72 Muratore CA, Sa LAB, Chiale PA, Eloy R, Tentori MC, Escudero J, Lima AMC, Medina LE, Garillo RI, Maloney J. Implantable cardioverter defibrillators and Chagas' disease: results of the ICD Registry Latin America. Europace (2009);11:164–168.

73 Rassi Jr A. Implantable Cardioverter-Defibrillators in Patients with Chagas Heart Disease: Misperceptions, Many Questions and the Urgent Need for a Randomized Clinical Trial. J Cardiovasc Electrophysiol 2007;:1-3.

74 Martinelli Filho M, Zimerman LI, Lorga AM, Vasconcelos JTM, Rassi A Jr. Guidelines for Implantable Electronic Cardiac Devices of the Brazilian Society of Cardiology. Arq Bras Cardiol 2007;89 (6):e210-e238.

75 Sternick EB, Martinelli M, Sampaio R, Gerken LM, Teixeira RA, Scarpelli R, Scanavacca M, Nishioka SD, Sosa E. Sudden cardiac death in patients with chagas heart disease and preserved left ventricular function. J Cardiovasc Electrophysiol. 2006;17(1):113-6.

76 http://www.finep.gov.br/transparencia/projeto_consolidado.asp?referencia=398705.

77 Volpe GJ, Trad HS, Koenigkam-Santos M, Moreira HT, Maciel BC, Marin-Neto JA, Schmidt A. Evaluation of Chagas heart disease by cardiac magnetic resonance after an aborted sudden cardiac death event. Journal of Cardiovascular Magnetic Resonance 2012 14(Suppl 1):P176.

78 Marin-Neto JA, Rassi A Jr, Maciel BC, Simões MV, Schmidt A. Chagas' heart disease . In :Yusuf S, Camm J , Fallen EL, Gersh BJ,editors. Evidence based cardiology. 3rd ed. London: BMJ Books; 2010.p.823-41.

79 O'Brien, P.C., Fleming, T. R. (1979) A multiple testing procedure for clinical trials. Biometrics, 35, 549-556.

80 Mathews. J. N. S. (2006) Introduction to Randomized Controlled Clinical Trials, Chapman & Hall/CRC.

81 Wilson JS, Podrid PJ. Side effects from amiodarone. Am Heart J. 1991;121(1 Pt 1):158-71.

82 Raeder EA, Podrid PJ, Lown B. Side effects and complications of amiodarone therapy. Am Heart J. 1985;109(5 Pt 1):975-83.

83 Freeman JV, Wang Y, Curtis JP, Heidenreich PA, Hlatky MA. The relation between hospital procedure volume and complications of cardioverter-defibrillator implantation from the implantable cardioverter-defibrillator registry. J Am Coll Cardiol. 2010;56(14):1133-9.

84 Lee DS, Krahn AD, Healey JS et al. Evaluation of early complications related to De Novo cardioverter defibrillator implantation insights from the Ontario ICD database. J Am Coll Cardiol. 2010;55(8):774-82.

Protocol, summary of changes

The changes between the first and last versions of the protocol are listed below:

1) The acceptable time window for recent positive serological tests for Chagas disease has been changed from the last 6 months to the last 2 years;
2) The threshold for renal dysfunction GFRe <60mL/min/1.73m2 was changed to GFRe <30mL/min/1.73m2;
3) In the most recent version, the refusal of patients with hypothyroidism was removed;
4) The latest version of the protocol defined the exclusion criteria for mental disorders, defining psychopathy or psychosis;
5) The definition of contraindication for the use of amiodarone (HR <55 bpm; sinus node disease; Mobitz type II; fixed AVB 2:1; advanced AVB; complete AVB; QTc >500msec), as well as the definition of formal indication for use of amiodarone or ICD (NSVT and very disturbing palpitations, presyncope or syncope; SVT; recovery from cardiac arrest);
6) The maximum limit for the maintenance dose of amiodarone increased from ≤100mg/day to ≤200mg/day;
7) The latest version already accepts the inclusion of patients with atrial fibrillation.

Note that NSVT stands for non-sustained ventricular tachycardia.

CHronic use of Amiodarone aGAinSt Implantable cardioverter-defibrillator therapy for primary prevention of death in patients with Chagas cardiomyopathy Study: Rationale and design of a randomized clinical trial

Martino Martinelli, MD, PhD,[a,h] Anis Rassi, Jr., MD, PhD,[b,h] José Antonio Marin-Neto, MD, PhD,[c,h] Angelo Amato Vincenzo de Paola, MD, PhD,[d,h] Otávio Berwanger, MD, PhD,[e,h] Maurício Ibraim Scanavacca, MD, PhD,[f,h] Roberto Kalil, MD, PhD,[g,h] and Sérgio Freitas de Siqueira, Eng, MSc[a,h]
São Paulo, and Goiânia, Brazil

Background The implantable cardioverter defibrillator (ICD) is better than antiarrhythmic drug therapy for the primary and secondary prevention of all-cause mortality and sudden cardiac death in patients with either coronary artery disease or idiopathic dilated cardiomyopathy. This study aims to assess whether the ICD also has this effect for primary prevention in chronic Chagas cardiomyopathy (CCC).

Methods In this randomized (concealed allocation) open-label trial, we aim to enroll up to 1,100 patients with CCC, a Rassi risk score for death prediction of ≥10 points, and at least 1 episode of nonsustained ventricular tachycardia on a 24-hour Holter monitoring. Patients from 28 centers in Brazil will be randomly assigned in a 1:1 ratio to receive an ICD or amiodarone (600 mg/d for 10 days, then 200-400 mg/d until the end of the study). The randomization sequence will be generated by computer, and the members of the committees responsible for end point validation and data analysis will be blinded to study assignment. The primary end point is all-cause death, and enrolment will continue until 256 patients have reached this end point. Key secondary end points include cardiovascular death, sudden cardiac death, hospitalization for heart failure, and quality of life. We expect follow-up to last 3 to 6 years, and data analysis will be done on an intention-to-treat basis. This trial is registered with ClinicalTrials.gov number NCT01722942.

Conclusion CHAGASICS is the first large-scale trial to assess the benefit of ICD therapy for the primary prevention of death in patients with CCC and nonsustained ventricular tachycardia, who have a moderate to high risk of death. (Am Heart J 2013;166:976-982.e4.)

Background

Chagas disease (American trypanosomiasis) is caused by the protozoan parasite *Trypanosoma cruzi* and is usually transmitted to humans through the feces of a bloodsucking insect of the Triatominae subfamily.[1,2] Infection often occurs during childhood, and the disease is characterized by 2 successive phases: acute and chronic. The chronic phase is classically divided into 4 main forms: indeterminate, cardiac, digestive, and cardiodigestive.[3] The heart is the most frequently and severely involved organ in the chronic phase. Up to 30% to 40% of patients with Chagas disease will ultimately have some degree of clinical cardiac involvement, which is the leading cause of cardiovascular death in areas where the disease is endemic.[4] Three syndromes are

From the [a]Pacemaker Clinic, Instituto do Coração (InCor), Universidade de São Paulo, São Paulo, Brazil, [b]Cardiology Division, Anis Rassi Hospital, Goiânia Brazil, [c]Cardiology Division, Faculdade de Medicina de Ribeirão Preto, Universidade de São Paulo, Ribeirão Preto, São Paulo, Brazil, [d]Cardiology Division, Escola Paulista de Medicina-UNIFESP, São Paulo, Brazil, [e]Research Institute HCor–Hospital do Coração, São Paulo, Brazil, [f]Arrhythmia Clinic, Instituto do Coração (InCor), Universidade de São Paulo, São Paulo, Brazil, and [g]Clinical Director, Instituto do Coração (InCor), Universidade de São Paulo, São Paulo, Brazil.
[h]On behalf of the CHAGASICS Investigators, Brazil.
Clinical trial registration: ClinicalTrials.gov no. NCT01722942.
Submitted May 16, 2013; accepted August 31, 2013.
Reprint requests: Martino Martinelli, MD, PhD, Clínica de Marcapasso do InCor-HC/FMUSP, Av. Enéas de Carvalho Aguiar, 44, São Paulo-SP 05403-000-Brazil.
E-mail: martino@incor.usp.br
0002-8703/$ - see front matter
© 2013, Mosby, Inc. All rights reserved.
http://dx.doi.org/10.1016/j.ahj.2013.08.027

characteristic of chronic Chagas cardiomyopathy (CCC)—arrhythmic syndrome, heart failure, and thromboembolic syndrome—which may occur alone or in association.[4]

It is estimated that 8 to 10 million people are currently infected with *T cruzi* in Latin America and nonendemic countries.[5,6] Based on this projection, 3 to 4 million individuals will presumably develop the cardiac or cardiodigestive forms of the disease in the chronic phase.

Despite some improvement in prognosis over the last years for patients with CCC,[7] sudden cardiac death (SCD), which is responsible for approximately 55% to 65% of all deaths in this disease[8], remains a major challenge. We estimate that at least 10,000 people with Chagas disease die from SCD annually, and approximately 90% of these events are caused by ventricular fibrillation.[9] Amiodarone or an implantable cardioverter defibrillator (ICD) or its association has been empirically used to prevent SCD in patients with CCC.[8] The ICD has been shown to be the most effective treatment for both primary and secondary prevention of arrhythmic death in clinical trials of patients who have had an episode of sustained ventricular tachycardia or survived after cardiac arrest[10] and also in clinical trials of patients who have severe left ventricular dysfunction and either coronary artery disease or idiopathic dilated cardiomyopathy.[11,12] However, only few observational studies and no randomized trials have examined whether the ICD is better than antiarrhythmic drug therapy for the primary (and even for the secondary) prevention of SCD in patients with CCC.[13]

The main hypothesis of this open-label randomized trial is that ICD implantation is more effective than drug therapy with amiodarone for the primary prevention of all-cause mortality in CCC patients with documented nonsustained ventricular tachycardia (NSVT), who are at moderate to high risk of death by the Rassi score.[14]

Methods

The CHAGASICS trial is registered with ClinicalTrials.gov number NCT01722942.

Financial support for this study is provided by grants from St Jude Medical Brazil Ltda and from FAPESP, a public taxpayer-funded foundation in the State of São Paulo and one of the main funding agencies for scientific and technological research in Brazil. These grants will cover usual costs associated with conducting the trial, such as data collection and management, research physician and nurse time, analysis of results, and clinical laboratory tests. The trial is also supported by the Brazilian Ministry of Health that will provide the ICDs and amiodarone. Implantable cardioverter defibrillator payments will be made directly to the hospitals participating in the trial by the Brazilian Unified Health System (Sistema Único de Saúde), in the same way it is done in other (eg, clinically indicated) circumstances.

The organizational structure for the trial is outlined in online Appendix A.

Patients

Patients from 28 centers in Brazil will be enrolled starting in September 2013. Completion of study enrollment is targeted for September 2016. Allowing for minimum 3-year follow-up of the final randomized patient, the trial should be completed by September 2019. Figure 1 shows the study design.

Male and female patients are eligible for the study if they meet the following inclusion criteria: 18 to 75 years of age; documented positive serological test for Chagas disease by at least 2 different methods (indirect hemagglutination, indirect immunofluorescence, or enzyme-linked immunosorbent assay) in the past 6 months; death risk score of Rassi[14] of at least 10 points; and at least 1 episode of NSVT on 24-hour Holter monitoring, which is defined as at least 3 successive beats (duration of <30 seconds), with a heart rate at least 120 beats/min. The exclusion criteria are provided in online Appendix B.

The Rassi score[14] stratifies patients with CCC into groups of low, moderate, and high risk of death from a combination of 6 risk markers (Figure 2): New York Heart Association (NYHA) functional class III or IV, cardiomegaly on chest x-ray, impaired left ventricular systolic function by echocardiography, NSVT on 24-hour Holter monitoring, low QRS voltage, and male gender.[14] The risk of death substantially increases when the Rassi score is ≥10. This score has been validated successfully in 2 external cohorts.[14,15]

To check whether patients meet the eligibility criteria, investigators will do a baseline reference examination regarding the essential information outlined above on all consecutive patients from each participating center who agree to participate in the trial (online Appendix C).

CHAGASICS adheres fully to the ethical principles of the Declaration of Helsinki, the specifications of the International Conference on Harmonization, and Good Clinical Practice fundamentals. The trial has been approved by each center's ethics committee, and every patient who agrees to participate in the trial will provide written informed consent.

Randomization, allocation concealment, and blinding

Eligible patients will be randomly assigned in a 1:1 ratio to receive an ICD or amiodarone immediately after the baseline reference examination. The randomization sequence will be computer generated (Interactive Web Response System), and allocation concealment will be ensured by the same system. This trial is open-label, so both patients and investigators will know which treatment groups patients have been assigned to. However, the Endpoint Validation Committee and the Data and Safety Monitoring Board (DSMB) will be blinded regarding this information.

Procedures

Patients assigned to the ICD group will be implanted with an ICD (St Jude Medical, Inc, Sylmar, CA) as soon as possible after randomization. It is recommended that all centers use single-chamber devices. The ICDs will be programmed to back up pacing in a ventricular inhibited mode at a rate of 40 beats/min and to detect ventricular fibrillation at 180 beats/min that will be treated with maximum shock energy. Antitachycardia pacing will be turned on to deliver bursts of 8 beats that begin at 81% of the tachycardia cycle length for tachycardia cycles below the ventricular fibrillation threshold; the bursts can be repeated at

Figure 1

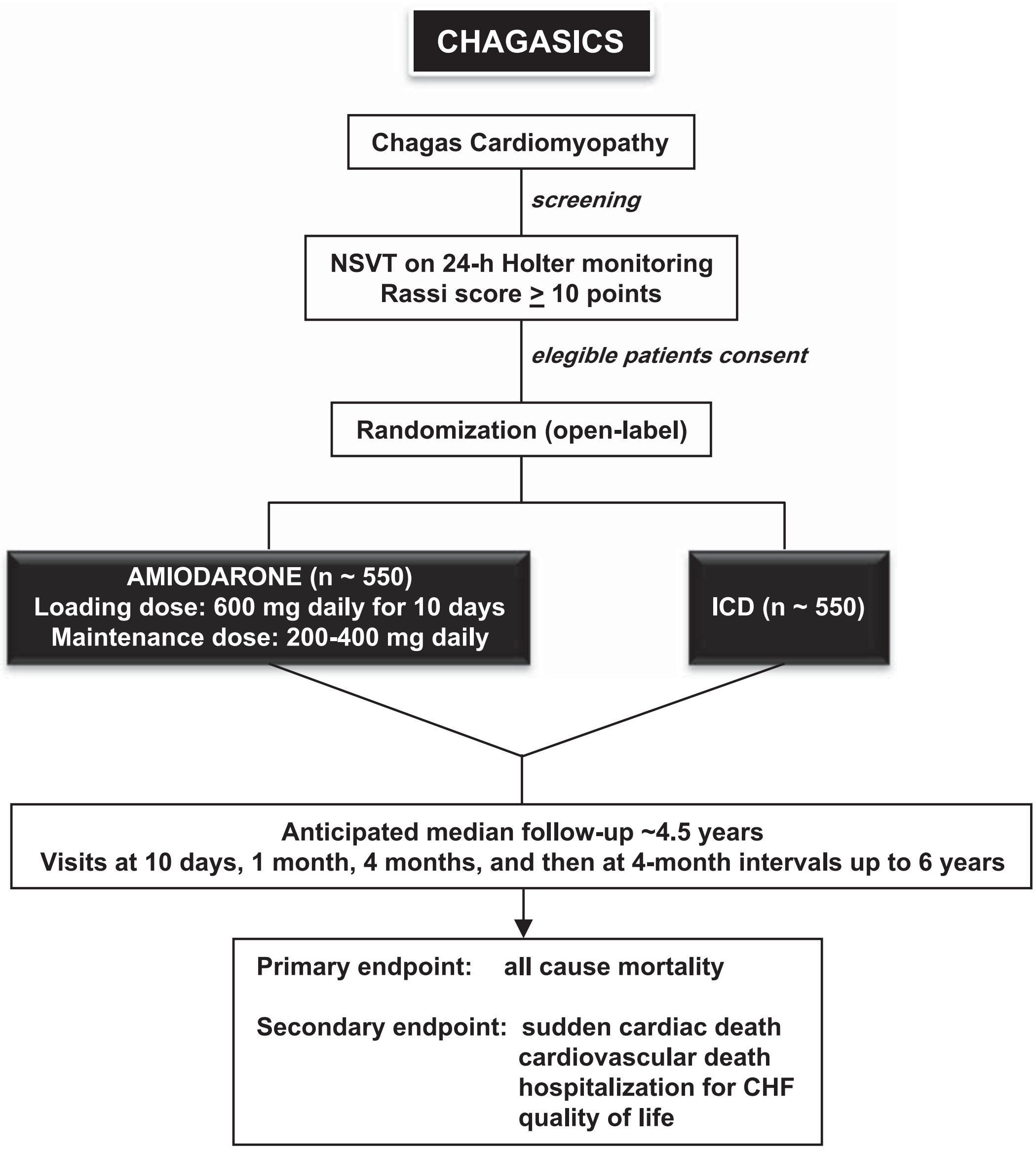

Study design of the CHAGASICS. *CHF*, Congestive heart failure.

the investigator's discretion only in cases of sustained ventricular tachycardia (with back-up shock therapy). St Jude Medical will provide technical support for assessment of the implanted devices. In the ICD group, use of amiodarone will be avoided, except in cases of multiple ICD shocks refractory to β-blockers (including sotalol) or catheter ablation, or both.

Patients randomly assigned to the amiodarone group will receive amiodarone hydrochloride (once a day) according to the following regimen: initial oral loading dose of 600 mg/d for 10 days on an outpatient basis, followed by an oral maintenance single dose of 200 to 400 mg/d until the end of the study. The optimum maintenance dose for each patient will be at the investigator's discretion and can be based on antiarrhthymic efficacy on 24-hour Holter monitoring, resting heart rate, side effects, and prolonged QTc interval. Dose adjustments will be allowed throughout the study period provided that the maintenance dose is kept at 200 to 400 mg/d. If the patient cannot tolerate a daily dose of 200 mg, amiodarone will be discontinued permanently, and treatment will be regarded as interrupted. If severe bradycardia or high-grade atrioventricular (AV) block occurs, a pacemaker will be implanted, and the amiodarone therapy will continue as planned. If the patient develops sustained ventricular arrhythmias or ventricular fibrillation, they could be treated with an ICD, but only as a last resort.

Figure 2

A

Risk factor	points
NYHA class III or IV	5
Cardiomegaly (chest X-ray)	5
Segmental or global WMA (2D echo)	3
Non-sustained ventricular tachycardia (24-h Holter)	3
Low QRS voltage (ECG)	2
Male sex	2

B

	Total mortality		
Total points	5 years	10 years	Risk
0–6	2%	10%	Low
7–11	18%	44%	Intermediate
12–20	63%	84%	High

Rassi score for prediction of death in patients with CCC. **A**, Independent prognostic factors and respective score points. **B**, Risk groups and total mortality at 5 and 10 years. *WMA*, Wall motion abnormality; *Echo*, transthoracic echocardiography; *ECG*, electrocardiography. Reproduced from *Lancet* 2010;375:1388-402[1] with permission of Rassi et al.

Although crossover in either direction is strongly discouraged, it is permitted when it is clearly in the patient's best interest.

Patients in both study groups will receive the best current medical drug therapy. Attending clinician investigators will be encouraged to treat study patients with angiotensin-converting enzyme inhibitors (or angiotensin II receptor blockers), spironolactone, acetylsalicylic acid, diuretics, and oral anticoagulants, where appropriate. Regarding β-blockers, although anecdotal observation suggests that their use may improve survival in patients with CCC,[16] the evidence is insufficient to make β-blocker therapy mandatory in this trial.[17,18] Disappointingly, the only randomized double-blind placebo controlled trial[19] that investigated the effect of a β-blocker (bisoprolol) on survival and hospitalization rates in patients with heart failure secondary to CCC, for unknown reasons has never been published. Nevertheless, to avoid disparities in β-blocker use between the 2 study groups, it is recommended that enrolling sites decide whether to use β-blockers in their patients before enrollment starts and a consistent approach be followed therein. Investigators should be aware that concomitant β-blocker and amiodarone therapy may be problematic in some patients due to an increased risk of developing or aggravating sinus node dysfunction or AV blocks. Patients should not be given any other antiarrhythmic drugs (with the exception of sotalol). Finally, trypanocidal therapy with benznidazole is left at the discretion of the attending clinical investigator responsible for each patient in the trial.

Follow-up

All patients will return for follow-up visits at 10 to 15 days, 1 month, and 4 months after randomization/implant of ICD/initiation of amiodarone and then at 4-month intervals until death or the end of the trial (up to 6 years after randomization). We expect median follow-up to last about 4.5 years. Unless the study is stopped prematurely, we assume that the temporal inclusion rate will be fairly constant during the expected inclusion period of 3 years and that all patients will have a minimum follow-up of 3 years. Online Appendix C shows the assessments that will be performed at each visit. Patients can be seen more frequently if needed. To avoid loss to follow-up, study nurses will attempt to contact each patient by telephone at least every 2 months.

Study end points

The primary end point is all-cause mortality. The local clinical investigators will be responsible for finding out if patients have died and immediately reporting deaths to the data coordinating center, even if supporting documents are not yet available. Investigators have to check whether patients who fail to attend scheduled follow-up visits are alive. The end point and other event validation committee, which will be blinded to treatment assignment, will categorize causes of death based on available information from witnesses, relatives and family members, death certificates, hospital records, autopsy reports, and device interrogation when applicable. The categories will be SCD, non-SCD, noncardiac death, and unknown. Deaths occurring after randomization but before the start of therapy will be included in the primary analysis according to the intention-to-treat principle. Perioperative deaths associated with implantation of the ICD will be regarded as non-SCD.

Secondary end points are specific mortality (SCD and cardiovascular death), hospital admission for new onset or worsening of heart failure, quality of life, need for back-up pacemaker implant (or pacing) in cases of severe bradycardia, and occurrence of defibrillator shocks (appropriate and not appropriate) in patients with an ICD implanted. We also aim to identify subsets of patients who have either no benefit or a substantial benefit from ICD therapy.

Statistical analysis

The primary analysis will compare time to all-cause death in the 2 study groups. We will estimate survival curves with the Kaplan-Meier method and use the log-rank test to assess the difference between ICD therapy and amiodarone. All randomized patients will be included in analyses, and they will be assessed according to their assigned treatment group irrespective of crossover or noncompliance (intention-to-treat principle). However, patient crossover in any direction will reduce the power of the study and could compromise the validity of the analysis. Thus, if crossover exceeds 20%, we will also analyze data using an “on-treatment” approach.

We will censor patients who are alive at the end of the follow-up period or who are lost to follow-up. Patients lost to follow-up will be censored at the time of last contact. We will use the Cox proportional hazards model to investigate important covariates from subgroup analysis regarding age, gender, NYHA functional class, Rassi score, and left ventricular ejection fraction. Similar statistical methods will be used for the secondary end points.

We calculated the sample size based on an expected mortality of 30% in patients given amiodarone after a mean follow-up of 4.5 years and an expected relative reduction in mortality of at least 30% (to ≤21%) in patients given an ICD. Allowing for loss to follow-up of up to 10%, we need approximately 1,100 patients (550 in each group) to achieve 90% power to detect the predicted difference in mortality between the treatment groups at a significance level of 0.05 (2 sided). To ensure we have enrolled enough patients to give 90% statistical power, enrollment will continue until at least 256 patients reach the primary end point. If this target is reached with <1,100 patients, the study can be stopped.

This sample size calculation also includes 2 interim analyses when a third and two-thirds of patients have reached the primary end point. The DSMB will be responsible for the interim analyses and may recommend interruption of the study for safety, benefit, or futility reasons. The committee will use the O’Brien-Fleming method, which considers different significance levels for each interim analysis, adopting extremely conservative values in the beginning of the study, when the number of events is small, and values closer to the pre-established level by the end of the study.[20,21]

Adverse events and criteria for discontinuation of amiodarone

Adverse events will be reported periodically to the data coordinating center. Preplanned procedures and preexisting disorders recorded at enrollment will not be regarded as adverse events unless a preexisting disorder worsens.

Adverse events will be classified according to their severity, cause, outcome, and relation with the study therapy. In the amiodarone group, the following disorders will be regarded as adverse events: proarrhythmia, thyroid disorders, liver toxicity, corneal or skin deposits, neuropathy due to accumulation of amiodarone, gastrointestinal disorders, and pulmonary fibrosis. Although there have been some concerns about the long-term toxicity of amiodarone, according to the author's experience, the use of low to moderate doses of this agent in patients with CCC is reasonably well tolerated for prolonged periods.

In the ICD group, the following disorders will be regarded as adverse events: infection at the implantation site of the generator or the leads and disorders related to the generator or leads malfunction, including inappropriate therapy. All serious adverse events, including primary and secondary end points, will have to be reported to the data coordinating center and to the local research ethics committee within 24 hours of occurrence. Online Appendix D outlines the criteria for discontinuation of amiodarone.

The authors have the overall responsibility for the design and conduct of this trial as well as for all study analysis and drafting and editing of the manuscript.

Discussion

No convincing data have as yet shown that the ICD is effective for primary or secondary prevention of death in CCC; the scientific evidence is restricted to single-center reports[22-25] or registries from ICD manufacturers,[26,27] with conflicting results.[13] Despite this lack of evidence, there is a relatively disseminated perception that it would be difficult to justify carrying out a large-scale randomized trial in patients with sustained life-threatening ventricular tachyarrhythmia because of obstacles concerning the use of a therapeutic alternative to the ICD. For this reason, our option was to conduct a primary prevention trial.

In CHAGASICS, we used eligibility criteria that ensured the ICD will be tested in patients at moderate to high death risk. Findings of recent studies[14,28] have shown that NSVT is an effective independent death predictor in CCC. Episodes of NSVT are a common finding on 24-hour Holter monitoring and usually have a high correlation with the severity of ventricular dysfunction.[29] Nonsustained ventricular tachycardia is seen in approximately 40% of patients with mild wall motion abnormalities on echocardiography and in almost 90% of patients with severe left ventricular dysfunction or heart failure, which is more frequent than in other cardiomyopathies.[29] However, we used both NSVT on 24-hour Holter monitoring and a death risk score of Rassi of at least 10 points as criteria for enrollment to ensure that study patients have a moderate to high risk of arrhythmic death.

Although left ventricular ejection fraction is not part of the Rassi score because, an echocardiogram is being performed to identify segmental or global wall motion abnormalities, it will be measured by Simpson method in all patients for a planned subgroup analysis. In contrast to patients with coronary artery disease or idiopathic dilated cardiomyopathy (in whom life-threatening arrhythmia is usually associated with reduced left ventricular ejection

fraction), in CCC, many patients develop malignant ventricular arrhythmias despite well-preserved global left ventricular systolic function. Therefore, this parameter will not be incorporated as an enrollment criterion.

Three randomized trials support the use of amiodarone in patients with NSVT: GESICA[30] (n = 516, 9.5% with Chagas disease), EPAMSA[31] (n = 127, 22% with Chagas disease), and AMIOVIRT[32] (n = 103, no patient with Chagas disease). Amiodarone was compared with a control group (no amiodarone) in GESICA and with placebo in EPAMSA, the only 2 randomized studies that included patients with Chagas disease. It significantly reduced overall mortality after a mean follow-up of 12 and 13 months, respectively. Of note, in the 2 trials, 80% to 90% of patients had complex ventricular arrhythmias (couplets or NSVT episodes, or both). In GESICA, amiodarone had a greater benefit in patients with NSVT at baseline (mortality reduced from 57% to 44%) than in those without NSVT (mortality reduced from 34% to 28%). In AMIOVIRT, the only randomized primary prevention trial of patients with dilated cardiomyopathy (non-Chagas) and NSVT at baseline, mortality did not differ between the ICD and amiodarone groups.

In addition to potentially reducing mortality, amiodarone is highly effective in suppressing ventricular ectopic beats,[33] with a low occurrence of severe side effects[34,35] and proarrhythmia,[36] particularly when given at low doses, in the range used in this study. Besides being a Singh-Vaughan Williams class III antiarrhythmic drug, amiodarone has a β-adrenergic blocker effect (class II) in animals stimulated by isoproterenol.[37,38] Furthermore, amiodarone rarely affects cardiac inotropism in a clinically significant manner, so it can be used even in patients with severe impairment of left ventricular systolic function.[30,39]

In the SCD-HeFT trial,[40] 2,521 patients with ischemic heart disease or idiopathic dilated cardiomyopathy, whose ejection fraction was 35% or lower, were given amiodarone, an ICD, or placebo. The findings, which show that amiodarone is no better than placebo for primary prevention of death in those patients, should not be extrapolated to patients with CCC. Nonsustained ventricular tachycardia (present in 23% of patients on 24-hour Holter monitoring at baseline) was not an inclusion criterion, and most patients were on regular optimized use of β-blockers. Moreover, although innumerous subgroup analyses were done, the authors did not show what we judge to be the most important analysis—a comparison of amiodarone with placebo in patients with and without NSVT.

We will not include a placebo group (treated with standard drug therapy but no ICD or antiarrhythmic medication) in the CHAGASICS because the aim of the study is the comparison of 2 acceptable therapies. In addition, that would double the sample size required, making the study unfeasible. Furthermore, with few exceptions, the findings of previous studies have not shown that amiodarone or the ICD harms the survival of patients with ischemic or nonischemic cardiomyopathy. Thus, it is plausible to assume that any observed difference in survival between the 2 groups would not be due to negative effects of one of the treatments, but rather due to a positive effect of the therapy. If mortality rates turn out to be equivalent between the treatment groups, then the questions would be whether both therapies are neutral or equally effective. By interrogating the ICDs and comparing the arrhythmia-free survival curves of both groups, we assume that it will be possible to discriminate between equally effective and similarly ineffective therapies.

Conclusions

The CHAGASICS will be the first study to assess whether a prophylactic ICD will reduce the mortality rate, compared with amiodarone, in patients with CCC and NSVT who are at moderate to high risk for death. Selection of eligible patients for therapy will be based on the Rassi score, and so the trial will help to establish the validity of this noninvasive strategy of risk stratification in this specific and sizable group. If the ICD improves survival, it will become a valuable additive treatment in these patients.

Acknowledgements

We thank Katrina Phillips for her careful review and editing of the text.

Disclosures

Dr Martinelli and Dr Scanavacca have received lecture fees from St Jude; Eng Siqueira was an employee of St Jude Medical Brasil Ltda; Dr Rassi, Jr, Dr Marin-Neto, Dr De Paola, Dr Berwanger, and Dr Kalil have no disclosures to declare.

References

1. Rassi Jr A, Rassi A, Marin-Neto JA. Chagas disease. Lancet 2010;375:1388-402.
2. Rassi Jr A, Rassi A, Marcondes de Rezende J. American trypanosomiasis (Chagas disease). Infect Dis Clin North Am 2012;26:275-91.
3. Rassi A, Rezende JM, Luquetti AO, et al. Clinical phases and forms of Chagas disease. In: Telleria J, Tibayrenc M, eds. American trypanosomiasis (Chagas disease). One hundred years of research. 1st edition. Burlington: Elsevier Inc.; 2010. p. 709-41.
4. Rassi Jr A, Rassi A, Little WC. Chagas' heart disease. Clin Cardiol 2000;23:883-9.
5. Salvatella R. Organizacion Panamericana de la Salud. Estimacion cuantitativa de la enfermedad de Chagas en las Americas. Montevideo, Uruguay: Organizacion Panamericana de la Salud, Report no. OPS/ HDM/CD/425-06. 2006 [in Spanish].

6. WHO. Chagas disease (American trypanosomiasis) fact sheet (revised in June 2010). Wkly Epidemiol Rec 2010;85:334-6.
7. Martins-Melo FR, Alencar CH, Ramos Jr AN, et al. Epidemiology of mortality related to Chagas' disease in Brazil, 1999-2007. PLoS Negl Trop Dis 2012;6:e1508.
8. Rassi Jr A, Rassi SG, Rassi A. Sudden death in Chagas' disease. Arq Bras Cardiol 2001;76:75-96.
9. Mendoza I, Moleiro F, Marques J. Sudden death in Chagas' disease. [Article in Portuguese]. Arq Bras Cardiol 1992;59:3-4.
10. Connolly SJ, Hallstrom AP, Cappato R, et al. Meta-analysis of the implantable cardioverter defibrillator secondary prevention trials. AVID, CASH and CIDS studies. Antiarrhythmics vs Implantable Defibrillator study. Cardiac Arrest Study Hamburg. Canadian Implantable Defibrillator Study. Eur Heart J 2000;21:2071-8.
11. Nanthakumar K, Epstein AE, Kay GN, et al. Prophylactic implantable cardioverter-defibrillator therapy in patients with left ventricular systolic dysfunction: a pooled analysis of 10 primary prevention trials. J Am Coll Cardiol 2004;44:2166-72.
12. Ezekowitz JA, Rowe BH, Dryden DM, et al. Systematic review: implantable cardioverter defibrillators for adults with left ventricular systolic dysfunction. Ann Intern Med 2007;147:251-62.
13. Rassi Jr A. Implantable cardioverter-defibrillators in patients with Chagas heart disease: misperceptions, many questions and the urgent need for a randomized clinical trial. J Cardiovasc Electrophysiol 2007;18:1241-3.
14. Rassi Jr A, Rassi A, Little WC, et al. Development and validation of a risk score for predicting mortality in Chagas' heart disease. N Engl J Med 2006;355:799-808.
15. Rocha MO, Ribeiro AL. A risk score for predicting death in Chagas' heart disease. N Engl J Med 2006;355:2488-9.
16. Issa VS, Amaral AF, Cruz FD, et al. Beta-blocker therapy and mortality of patients with Chagas cardiomyopathy: a subanalysis of the REMADHE prospective trial. Circ Heart Fail 2010;3:82-8.
17. Rassi Jr A, Rassi A. Letter by Rassi et al regarding article, "Beta-blocker therapy and mortality of patients with Chagas cardiomyopathy: a subanalysis of the REMADHE prospective trial". Circ Heart Fail 2010;3:e11.
18. Hidalgo R, Martí-Carvajal AJ, Kwong JS, et al. Pharmacological interventions for treating heart failure in patients with chagas cardiomyopathy. Cochrane Database Syst Rev 2012 Nov 14;11: CD009077.
19. Quiros FR, Morillo CA, Casas JP, et al. CHARITY: Chagas cardiomyopathy bisoprolol intervention study: a randomized double-blind placebo force-titration controlled study with Bisoprolol in patients with chronic heart failure secondary to Chagas cardiomyopathy [NCT00323973]. Trials 2006;7:21.
20. O'Brien PC, Fleming TR. A multiple testing procedure for clinical trials. Biometrics 1979;35:549-56.
21. Mathews JNS. Introduction to randomized controlled clinical trials. Chapman & Hall/CRC. 2006.
22. Cardinalli-Neto A, Bestetti RB, Cordeiro JA, et al. Predictors of all-cause mortality for patients with chronic Chagas' heart disease receiving implantable cardioverter defibrillator therapy. J Cardiovasc Electrophysiol 2007;18:1236-40.
23. Cardinalli-Neto A, Nakazone MA, Grassi LV, et al. Implantable cardioverter-defibrillator therapy for primary prevention of sudden cardiac death in patients with severe Chagas cardiomyopathy. Int J Cardiol 2011;150:94-5.
24. Martinelli M, Siqueira SF, Sternick EB, et al. Long-term follow-up of implantable cardioverter-defibrillator for secondary prevention in Chagas' heart disease. Am J Cardiol 2012;110:1040-5.
25. Barbosa MP, da Costa Rocha MO, de Oliveira AB, et al. Efficacy and safety of implantable cardioverter-defibrillators in patients with Chagas disease. Europace 2013;15:957-62.
26. Garillo R, Greco OT, Oseroff O, et al. Cardioverter defibrillator implantable as a secondary prevention in the Chagas' disease. The results of the Latin-American Studies ICD LABOR. Reblampa 2004; 17. [article in Spanish].
27. Muratore CA, Batista Sa LA, Chiale PA, et al. Implantable cardioverter defibrillators and Chagas' disease: results of the ICD Registry Latin America. Europace 2009;11:164-8.
28. Ribeiro AL, Cavalvanti PS, Lombardi F, et al. Prognostic value of signal-averaged electrocardiogram in Chagas disease. J Cardiovasc Electrophysiol 2008;19:502-9.
29. Rassi Jr A, Gabriel Rassi A, Gabriel Rassi S, et al. Ventricular arrhythmia in Chagas disease. Diagnostic, prognostic, and therapeutic features. Arq Bras Cardiol 1995;65:377-87. [in Portuguese].
30. Doval HC, Nul DR, Grancelli HO, et al. Randomised trial of low-dose amiodarone in severe congestive heart failure. Grupo de Estudio de la Sobrevida en la Insuficiencia Cardiaca en Argentina (GESICA). Lancet 1994;344:493-8.
31. Garguichevich JJ, Ramos JL, Gambarte A, et al. Effect of amiodarone therapy on mortality in patients with left ventricular dysfunction and asymptomatic complex ventricular arrhythmias: Argentine Pilot Study of Sudden Death and Amiodarone (EPAMSA). Am Heart J 1995; 130:494-500.
32. Strickberger SA, Hummel JD, Bartlett TG, et al. for the AMIOVIRT Investigators. Amiodarone versus implantable cardioverter-defibrillator: randomized trial in patients with nonischemic dilated cardiomyopathy and asymptomatic nonsustained ventricular tachycardia—AMIOVIRT. J Am Coll Cardiol 2003;41:1707-12.
33. Chiale PA, Halpern MS, Nau GJ, et al. Efficacy of amiodarone during long-term treatment of malignant ventricular arrhythmias in patients with chronic chagasic myocarditis. Am Heart J 1984;107: 656-65.
34. Vorperian VR, Havighurst TC, Miller S, et al. Adverse effects of low dose amiodarone: a meta-analysis. J Am Coll Cardiol 1997;30: 791-8.
35. Sopher SM, Camm AJ. Adverse effects of amiodarone at low dose: plus ça change. J Am Coll Cardiol 1997;30:799-801.
36. Hohnloser SH, Klingenheben T, Singh BN. Amiodarone-associated proarrhythmic effects: a review with special reference to *torsade de pointes* tachycardia. Ann Intern Med 1994;121:529-35.
37. Harris L, Chatelain P, Nokin P, et al. Pharmacology of amiodarone. In Amiodarone. Pharmacology, pharmacokinetics, toxicology, clinical effects. In: Harris L, Roncucci R, eds. Paris: Médecine et Sciences Internationales; 1986. p. 23-4.
38. Singh BN. Antiarrhythmic actions of amiodarone: a profile of a paradoxical agent. Am J Cardiol 1996;78(4A):41-53.
39. Massie BM, Fisher SG, Radford M, et al. Effect of amiodarone on clinical status and left ventricular function in patients with congestive heart failure. CHF-STAT Investigators. Circulation 1996; 93:2128-34.
40. Bardy GH, Lee KL, Mark DB, et al. Amiodarone or an implantable cardioverter-defibrillator for congestive heart failure. N Engl J Med 2005;352:225-37.

Appendix A. Study organization

CRO, contract research organization. DSMB, data and safety monitoring board.

Study chair and principal investigator

- Martino Martinelli, São Paulo, SP.
- Project leader, responsible for all interactions among the committees.
- Final responsibility for the overall study, the budget, compliance, quality assurance, and data integrity.

Steering committee

- Martino Martinelli Filho, São Paulo, SP; Anis Rassi Jr, Goiânia, GO; José Antonio Marin-Neto, Ribeirão Preto, SP; Angelo Amato Vincenzo de Paola, São Paulo, SP; Otávio Berwanger, São Paulo, SP; Maurício Ibraim Scanavacca, São Paulo, SP; Roberto Kalil, São Paulo, SP; Sérgio Freitas de Siqueira, São Paulo, SP.
- Study chair + 7 investigators with clinical and methodological expertise in clinical trials, Chagas disease, or both.
- Responsible for the scientific direction, protocol design and amendments, policy decisions about sub studies, final analysis of all data (blinded to the identification of groups), monitoring progress of the study, reporting and publishing the trial results, selecting the sites participating in the trial, and selecting members of the executive, DSMB and end point validation committees.

Executive committee

- Composed of the study chair, members of the steering committee, 1 secretariat member, and the principal investigator at each clinical site.
- Responsible for the overall organization and conduct of the trial and ensuring that data are collected according to accepted practices.

Data coordinating center

- Located at the Instituto do Coração (InCor), Hospital das Clínicas da Faculdade de Medicina da USP, São Paulo, Brazil.
- Responsible, in conjunction with a CRO (Coreware, São Paulo, SP, Brazil), for randomization, electronic case report forms, data acquisition, data entry, data processing, reporting adverse events, amiodarone distribution to the investigation centers, and providing statistical reports and other requests to the DSMB.
- Data will be collected through the internet into a secure server at the CRO.

Independent DSMB

- José Carlos Nicolau, São Paulo, SP; Leopoldo Piegas, São Paulo, SP; Antônio Carlos Carvalho, São Paulo, SP; and an experienced biostatitician.
- Reviews safety data every 6 months and will notify the study chair if they have concerns about continuation of the study.
- Also responsible for the formal interim analysis of efficacy in accordance with the study protocol.

End point validation committee

- Adjudicates all major morbidity and mortality events, without knowledge of treatment group.

Clinical sites

- Each site will have at least a principal investigator (physician) and a clinical coordinator who will be actively involved in the recruitment, evaluation, and treatment of participants.
- Luiz Pereira de Magalhães, Hospital Ana Nery, Salvador, BA; Ricardo Eloy Pereira, Hospital Santa Izabel, Salvador, BA; Francisca Tatiana Moreira Pereira, Hospital Universitário Walter Cantideo, Fortaleza, CE; Stela Maria Vitorino Sampaio, Hospital de Messejana, Fortaleza, CE; José Mario Baggio Junior, Instituto de Cardiologia do Distrito Federal, Brasília, DF; Ayrton Klyer Peres, Hospital de Base do Distrito Federal, Brasília, DF; Sérgio Gabriel Rassi, Anis Rassi Hospital, Goiânia, GO; Salvador Rassi, Hospital das Clínicas de Goiânia, Goiânia, GO; Antonio Malan C. Lima, Santa Casa de Goiânia, Goiânia, GO; Henrique Barroso Moreira, Hospital das Clínicas da UFMG, Belo Horizonte, MG; Thiago da Rocha Rodrigues, Hospital Felício Rocho, Belo Horizonte, MG; Ricardo Alkmim Teixeira, Hospital das Clínicas Samuel Libânio, Pouso Alegre, MG; Celso Salgado de Melo, Hospital Escola da Universidade Federal do Triângulo Mineiro, Uberaba, MG; Júlio César de Oliveira, Hospital Geral Universitário, Cuiabá, MT; Dário Celestino Sobral Filho, Pronto Socorro Cardiológico de PE Prof. Luiz Tavares – PROCAPE, Recife, PE; Gerson Lemke, Hospital Santa Casa de Misericórdia de Curitiba, Curitiba, PR; Márcio Jansen de Oliveira Figueiredo, Hospital das Clínicas da UNICAMP, Campinas, SP; José Marco Nogueira Lima, Hospital e Maternidade Celso Pierro – PUCC, Campinas, SP; Rodrigo Tavares Silva, Hospital do Coração da Fundação de Misericórdia de Franca, Franca, SP; Marcelo Garcia Leal, Hospital das Clínicas – FMUSP/Ribeirão Preto, Ribeirão Preto, SP; Adalberto Lorga Filho, Instituto de Moléstias Cardiovasculares, São José do Rio Preto, SP; Juan Carlos Pachon Mateos and Abilio Fragata, Instituto Dante Pazzanese de Cardiologia, São Paulo, SP; Sylvio Matheus de Aquino Gandra, Irmandade da Santa Casa de Misericórdia de São Paulo, São Paulo, SP; Silas dos Santos Galvão Filho, Beneficência Portuguesa, São Paulo, SP; Antonio Vitor Moraes Júnior, Santa Casa de Ribeirão Preto, Ribeirão Preto, SP; Genildo Ferreira Nunes, Hospital Geral Publico de Palmas, Palmas, TO. Álvaro Sarabanda, Instituto de Cardiologia do Distrito Federal, Brasília, DF; Mauro

Esteves Hernandes, Santa Casa de Votuporanga, Votuporanga, SP.

Appendix B. Exclusion criteria

ICD, implantable cardioverter defibrillator. NSVT, non-sustained ventricular tachycardia. bpm, beats per min. AV, atrioventricular. NYHA, New York Heart Association.

- Exposure to amiodarone in the past 6 months, unless total dose is less than 10 g, exposure is for less than 2 weeks, or maintenance dose is 100 mg/d or lower
- Formal indication for the use of amiodarone or an ICD (NSVT with disturbing palpitations, presyncope or syncope; sustained ventricular tachycardia; previous cardiac arrest)
- Clearly identified transient or correctable cause for NSVT
- Liver function test results at more than three times the upper normal limit or a serum creatinine concentration of more than 221 μmol/L (2.5 mg/dL)
- Moderate or severe chronic obstructive pulmonary disease
- Peripheral polyneuropathy
- Hypothyroidism or hyperthyroidism or receiving therapy for these conditions
- Pregnancy, breastfeeding, or childbearing potential (ie, nonmenopausal women who are not using medically prescribed contraceptive measures)
- Other contraindications for the use of amiodarone: previous intolerance to the drug; sustained rest heart rate of less than 55 bpm; sinus node disease, type II second degree AV block, fixed 2:1 AV block, advanced degree AV block, or complete AV block, without pacemaker implantation; QTc of more than 500 ms
- Current use of other obligatory drug therapies with contraindication to the concomitant use of amiodarone
- Other concomitant cardiovascular disease, including uncontrolled diabetes mellitus (systemic hypertension without target organ damage is allowed)
- Unexplained syncope in the past year
- Current use of β-blocker considered clinically indispensable, with bradycardia at less than 55 bpm or AV block of more than first degree, without pacemaker implantation
- History of psychiatric disorders or active alcohol or drug abuse
- Severe systemic illness that is likely to be fatal during the course of the trial
- Marked hypotension; uncorrected hypokalemia
- Severe symptoms of heart failure (NYHA functional class IV) at the time of enrollment
- Persistent or permanent atrial fibrillation
- Poor compliance or any disorder that, in the opinion of the investigator, might prevent the patient from adhering to the trial protocol and scheduled visits
- Current participation or participation in the past year in any other therapeutic trial

Appendix C

Supplementary Table. Schedule of baseline and follow-up investigations

		Postrandomization (m)																			
	Baseline	10 d	1	4	8	12	16	20	24	28	32	36 min	40	44	48	52	56	60	64	68	72 max
Inclusion and exclusion criteria verified	X																				
Patient informed consent	X																				
Medical history	X	X	X	X	X	X	X	X	X	X	X	X	X	X	X	X	X	X	X	X	X
Physical examination	X	X	X	X	X	X	X	X	X	X	X	X	X	X	X	X	X	X	X	X	X
Changes in cardiovascular drug therapy	X	X	X	X	X	X	X	X	X	X	X	X	X	X	X	X	X	X	X	X	X
Vital status	X	X	X	X	X	X	X	X	X	X	X	X	X	X	X	X	X	X	X	X	X
NYHA class	X	X	X	X	X	X	X	X	X	X	X	X	X	X	X	X	X	X	X	X	X
12-lead ECG	X	X	X			X			X			X			X			X			X
Chest x-ray	X					X			X			X			X			X			X
24-h Holter	X	X		X		X															
2D echocardiography	X					X			X			X			X			X			X
Biochemical tests*	X		X			X			X			X			X			X			X
Quality of life questionnaire	X			X		X			X			X			X			X			X
ICD interrogation†		X	X	X	X	X	X	X	X	X	X	X	X	X	X	X	X	X	X	X	X
Compliance (pill count) and maintenance amiodarone dose‡		X	X	X	X	X	X	X	X	X	X	X	X	X	X	X	X	X	X	X	X
Adverse events		X	X	X	X	X	X	X	X	X	X	X	X	X	X	X	X	X	X	X	X

ECG, Electrocardiography. *ICD*, implantable cardioverter defibrillator.
*Biochemical blood analysis for complete blood count, creatinine, urea, alanine aminotransferase, aspartate aminotransferase, bilirubins, magnesium, potassium, thyroid-stimulating hormone, and free thyroxine.
†Only in patients in the ICD group.
‡Only in patients in the amiodarone group.

Appendix D. Clinical indicators for discontinuation of amiodarone

PVC, premature ventricular contraction.

Indicators for permanent discontinuation

- Proarrhythmia

-Increased density of PVCs on 24-h Holter monitoring

Baseline density (mean PVC per h)	Increase consistent with proarrhythmia
• 10-100	• 7×
• 101-300	• 4×
• 301-1000	• 3×
• >1000	• 2×

-Increase in NSVT

Baseline density (NVST per 24 h)	Increase consistent with proarrhythmia
• <5	• ≥50 episodes per 24 h
• ≥5	• >10×

-Torsade de pointes

- Liver abnormalities: increased bilirubins, aspartate aminotransferase, alanine aminotransferase, or alkaline phosphatase by at least 3 times the upper normal limit or by 2 to 3 times the upper normal limit for 2 months or longer
- Thyroid abnormalities: symptomatic hyperthyroidism
- Ophthalmic abnormalities: significant corneal microdeposits with blurred vision or perception of a colored halo around a light source when the patient looks at it
- Prolonged QTc interval (>550 ms) or QTc interval dispersion (>80 ms), or both

Indicators for discontinuation or dose reduction at the investigator's or the patient's discretion

- Neurological abnormalities: ataxia and hand tremor
- Skin abnormalities: blue-gray discoloration of the face or skin hyperchromia, or both
- Gastrointestinal abnormalities: nausea, vomiting, and constipation, or a combination

Recommended actions for other clinical indicators

- Increased TSH (asymptomatic): observe patient closely

- Symptomatic hypothyroidism: start L-thyroxine, continue amiodarone at the investigator's discretion
- New chest x-ray abnormalities (interstitial or diffuse bilateral alveolar infiltrate, or both) not explained by congestive heart failure or pulmonary infection: discontinue amiodarone treatment and do laboratory tests (eg, pulmonary gallium scintigraphy, bronchoscopy and lung biopsy), corticosteroids may be used, treatment may be resumed depending on test results
