# Supplementary material for: Amiodarone or Implantable Cardioverter-Defibrillator in Chagas Cardiomyopathy: The CHAGASICS Randomized Clinical Trial
Source: JAMA Cardiol. 2024 Oct 2;9(12):1073–81. doi: 10.1001/jamacardio.2024.3169 (PMC11447631; doi:10.1001/jamacardio.2024.3169)
Supplement: Supplement 2. — eTable 1. Amiodarone Use During Follow-Up eTable 2. Findings of Primary and Secondary End Points of the Study eTable 3. Appropriate and Inappropriate ICD Therapies eTable 4. Change in Left Ventricular Ejection Fraction During Follow-Up eTable 5. NYHA Functional Class During Follow-Up eTable 6. Adverse Events by Treatment Group eTable 7. Concomitant Use of Medication at the End of Follow-Up by Treatment Group [file jamacardiol-e243169-s002.pdf]

Supplementary Online Content

Martinelli-Filho M, Marin-Neto JA, Scanavacca MI, et al. Amiodarone or implantable cardioverter-defibrillator in Chagas cardiomyopathy: the CHAGASICS randomized clinical trial. *JAMA Cardiol*. Published online October 2, 2024. doi:10.1001/jamacardio.2024.3169

eTable 1. Amiodarone Use During Follow-Up

eTable 2. Findings of Primary and Secondary End Points of the Study

eTable 3. Appropriate and Inappropriate ICD Therapies

eTable 4. Change in Left Ventricular Ejection Fraction During Follow-Up

eTable 5. NYHA Functional Class During Follow-Up

eTable 6. Adverse Events by Treatment Group

eTable 7. Concomitant Use of Medication at the End of Follow-Up by Treatment Group

This supplementary material has been provided by the authors to give readers additional information about their work.

© 2024 American Medical Association. All rights reserved.

eTable 1. Amiodarone Use During Follow-Up

**Initial dose (mg/day), n (%)**	**Amiodarone (n=166)**
100	1(0,6)
200	89 (53.6)
300	15(9.0)
400	56 (33.7)
600	2 (1.2)
Missing (%)	3 (1.8)
**Mean amiodarone dose at the end of the study (mg/day), n (%)**	277,15mg
100 and <200	4 (2.4)
200 and <300	84 (50.6)
300 and <400	48 (28.9)
400 and <500	22 (13.3)
500 and <600	0 (0.0)
600	2(1.2)
Missing (%)	7 (4.2)

© 2024 American Medical Association. All rights reserved.

eTable 2. Findings Of Primary And Secondary End Points Of The Study

	ICD (n=157)		Amiodarone (n=166)		Difference in RMST (95% CI), days	*P*
	No. events	RMST, days	No. events	RMST, days		
**Primary outcome**						
All-cause death	60	1494	64	1390	104 (-22, 229)	.10
**Secondary outcomes**						
Cardiovascular death	46	1536	50	1432	104 (-22, 229)	.11
Heart failure hospitalization	22	1623	36	1476	148 (18, 277)	.03

ICD: implantable cardioverter–defibrillator; RMST: restricted mean survival time

© 2024 American Medical Association. All rights reserved.

eTable 3. Appropriate and Inappropriate ICD Therapies

	ICD (n=157)	
**ICD therapy**	**Events, n**	**Patients, n (%)**
**Antitachycardia Pacing Therapy**	118	57 (36.3)
Appropriate[a]	96	50 (31.8)
Inappropriate[b]	19	16 (10.2)
**Cardioversion/Defibrillation Shock Therapy**	81	56 (35.7)
Appropriate[a]	48	40 (25.5)
Inappropriate[b]	19	13 (8.3)

ICD: implantable cardioverter–defibrillator;

[a] - therapy delivered for rhythms considered to be ventricular tachycardia;

[b] - therapy delivered for rhythms not considered to be ventricular tachycardia.

© 2024 American Medical Association. All rights reserved.

eTable 4. Change in Left Ventricular Ejection Fraction During Follow-Up

	ICD (n=157)	Amiodarone (n=166)	Adjusted mean difference* (95% CI)	*P*
**1 year**				
Number of patients	99	87		
Change in LVEF from baseline, mean (SD)	-1.7 (10.1)	-1.3 (8.3)	-1.34 (-3.94, 1.27)	.31
**2 years**				
Number of patients	70	69		
Change in LVEF from baseline, mean (SD)	-2.0 (8.2)	-2.4 (8.6)	0.10 (-2.68, 2.88)	.94
**3 years**				
Number of patients	48	44		
Change in LVEF from baseline, mean (SD)	-1.8 (9.2)	-2.9 (10.2)	-0.62 (-4.44, 3.19)	.75

LVEF: left ventricular ejection fraction; SD: standard deviation

* Estimated using ANCOVA adjusting for baseline LVEF. A mean difference <0 means that patients in the ICD arm have a greater decrease in LVEF compared with patients in the amiodarone arm.

© 2024 American Medical Association. All rights reserved.

eTable 5. NYHA Functional Class During Follow-Up

	ICD (n=157)	Amiodarone (n=166)	Common OR* (95% CI)	P
**1 year**				
Number of patients	123	138		
NYHA, n (%)			0.49 (0.31, 0.79)	.003
I	57 (46.3)	42 (30.4)		
II	53 (43.1)	69 (50.0)		
III	7 (5.7)	10 (7.2)		
IV	2 (1.6)	1 (0.7)		
Dead	4 (3.3)	16 (11.6)		
**2 years**				
Number of patients	124	131		
NYHA, n (%)			0.47 (0.30, 0.75)	.002
I	52 (41.9)	33 (25.2)		
II	52 (41.9)	61 (46.6)		
III	7 (5.6)	9 (6.9)		
IV	0 (0.0)	1 (0.8)		
Dead	13 (10.5)	27 (20.6)		
**3 years**				
Number of patients	131	131		
NYHA, n (%)			0.57 (0.37, 0.89)	.01
I	42 (32.1)	27 (20.6)		
II	51 (38.9)	51 (38.9)		
III	10 (7.6)	10 (7.6)		
IV	0 (0.0)	1 (0.8)		
Dead	28 (21.4)	42 (32.1)		

© 2024 American Medical Association. All rights reserved.

* Estimated using an ordinal logistic regression model. A common OR <1 means that patients in the ICD arm have an improved NYHA class compared with patients in the amiodarone arm.

© 2024 American Medical Association. All rights reserved.

eTable 6. Adverse Events by Treatment Group

	ICD (n=157)<br>n (%)
**Related to ICD**	**4 (2.5)**
Pocket hematoma	1 (0.6)
Lead dysfunction	2 (1.3)
Pocket Infection	1 (0.6)
	**Amiodarone (n=166)<br>n (%)**
**Related to amiodarone**	**10 (6.0)**
Hypothyroidism	5 (2.4)
New thyroid nodule	1 (0.6)
Corneal deposits	1 (0.6)
Skin hyperpigmentation	1 (0.6)
Gastrointestinal	2 (1.2)

ICD: implantable cardioverter–defibrillator

© 2024 American Medical Association. All rights reserved.

eTable 7. Concomitant Use of Medication at the End of Follow-Up by Treatment Group

Medication	ICD (n=157)	Amiodarone (n=166)	*P*
ACE-i, n (%)	80 (24.8)	75 (23.2)	.29
Aldosterone antagonist, n (%)	21 (6.5)	27 (8.4)	.46
ARB, n (%)	42 (13.0)	38 (11.8)	.42
Beta-blocker, n (%)	123 (38.1)	118 (36.5)	.13
Calcium channel blocker, n (%)	4 (1.2)	6 (1.9)	.58
Digitalis, n (%)	11 (3.4)	10 (3.1)	.72
Diuretic, n (%)	108 (33.4)	102 (31.6)	.16
Nitrate, n (%)	3 (0.9)	1 (0.3)	.28
Hydralazine, n (%)	5 (1.5)	13 (4.0)	.06
Other antiarrhythmic, n (%)	3 (0.9)	1 (0.3)	.28
Other, n (%)	127 (39.3)	144 (44.6)	.15

ICD: implantable cardioverter–defibrillator; ACEi: angiotensin-converting enzyme inhibitor; ARB: angiotensin receptor blocker.

© 2024 American Medical Association. All rights reserved.
