# Supplementary material for: Amiodarone or Implantable Cardioverter-Defibrillator in Chagas Cardiomyopathy: The CHAGASICS Randomized Clinical Trial
Source: JAMA Cardiol. 2024 Oct 2;9(12):1073–81. doi: 10.1001/jamacardio.2024.3169 (PMC11447631; doi:10.1001/jamacardio.2024.3169)
Supplement: Supplement 3. — Nonauthor Collaborators. The CHAGASICS investigators [file jamacardiol-e243169-s003.pdf]

***First Name and Middle Initial(s)**	*Last Name	***Suffix (eg, Jr, III)**	Academic Degrees	Institution	Location (city, state/province, country)	Role or Contribution, eg, chair, principal investigator	Group (if more than 1 Group listed in the byline) and/or Subgroup (eg, Steering Committee)
Jose O. P.	Penteado		MD	Instituto Brandao de Reabilitacao	Vitoria da Conquista, BA, Brazil	Recruitment, monitoring, device implantation and patient data acquisition, participation in study meetings	CHAGASICS Investigators
Francisca T. M.	Pereira		MD	Hospital Universitário Walter Cantideo - Universidade Federal do Ceará	Fortaleza, CE, Brazil	Recruitment, monitoring, device implantation and patient data acquisition, participation in study meetings	CHAGASICS Investigators
Marcelo G.	Leal		MD	Department of Cardiology, Faculdade de Medicina de Ribeirão Preto, Universidade de São Paulo	Ribeirao Preto, SP, Brazil	Recruitment, follow-up and data aquisition, paticipation on meetings of the study	CHAGASICS Investigators
Salvador	Rassi		PhD, MD	Hospital das Clinicas da Universidade Federal de Goias	Goiania, GO, Brazil	Recruitment, follow-up and data aquisition, paticipation on meetings of the study	CHAGASICS Investigators
Fausto	Feres		Phd, MD	Department of Cardiology, Instituto Dante Pazzanese de Cardiologia (IDPC)	Sao Paulo, SP, Brazil	Local Center Coordinator	CHAGASICS Investigators
Cecilia M. B.	Barcellos		MD	Department of Cardiology, Instituto Dante Pazzanese de Cardiologia (IDPC)	Sao Paulo, SP, Brazil	Recruitment, monitoring, device implantation and patient data acquisition, participation in study meetings	CHAGASICS Investigators
Adalberto M.	Lorga	Filho	PhD, MD	Instituto de Moléstias Cardiovasculares - IMC	Sao Jose do Rio Preto, SP, Brazil	Recruitment, monitoring, device implantation and patient data acquisition, participation in study meetings	CHAGASICS Investigators
Carlos E. B.	Lima		Phd, MD	Universidade Federal Do Piaui - UFPI	Teresina, PI, Brazil	Recruitment, monitoring, device implantation and patient data acquisition, participation in study meetings	CHAGASICS Investigators
Alvaro V. L.	Sarabanda		PhD, MD	Instituto de Cardiologia do Distrito Federal - ICDF	Brasilia, DF, Brazil	Recruitment, monitoring, device implantation and patient data acquisition, participation in study meetings	CHAGASICS Investigators
José M.	Baggio	Jr	MD	Instituto de Cardiologia do Distrito Federal - ICDF	Brasilia, DF, Brazil	Recruitment, monitoring, device implantation and patient data acquisition, participation in study meetings	CHAGASICS Investigators
Lilia N.	Maia		PhD, MD	Hospital de Base da Fundacao Faculdade Regional de Medicina de Sao Jose do Rio Preto (HB/FUNFARME)	Sao Jose do Rio Preto, SP, Brazil	Recruitment, follow-up and data aquisition, paticipation on meetings of the study	CHAGASICS Investigators
Roberto	Costa		PhD, MD	Department of Cardiovascular Surgery, Instituto do Coração (InCor), Hospital das Clínicas da Universidade de São Paulo	Sao Paulo, SP, Brazil	Device implantation, participation in study meetings	CHAGASICS Investigators
Dariana V. A.	Penteado		MD	Instituto Brandao de Reabilitacao	Vitoria da Conquista, BA, Brazil	Recruitment, follow-up and data aquisition, paticipation on meetings of the study	CHAGASICS Investigators
Anísio A. A.	Pedrosa		PhD, MD	Department of Cardiology, Instituto do Coração (InCor), Hospital das Clínicas da Universidade de São Paulo	Sao Paulo, SP, Brazil	Follow-up and data aquisition, paticipation on meetings of the study	CHAGASICS Investigators
Caio V.	Spiaggiari		PhD, MD	Department of Cardiology, Instituto do Coração (InCor), Hospital das Clínicas da Universidade de São Paulo	Sao Paulo, SP, Brazil	Follow-up and data aquisition, paticipation on meetings of the study	CHAGASICS Investigators
Cinthya I. G.	Gomes		MD	Department of Cardiology, Instituto do Coração (InCor), Hospital das Clínicas da Universidade de São Paulo	Sao Paulo, SP, Brazil	Follow-up and data aquisition, paticipation on meetings of the study	CHAGASICS Investigators
Marcos G. M.	Saccab		MD	Department of Cardiology, Instituto do Coração (InCor), Hospital das Clínicas da Universidade de São Paulo	Sao Paulo, SP, Brazil	Follow-up and data aquisition, paticipation on meetings of the study	CHAGASICS Investigators
Ricardo A.	Teixeira		PhD, MD	Department of Cardiology, Instituto do Coração (InCor), Hospital das Clínicas da Universidade de São Paulo	Sao Paulo, SP, Brazil	Follow-up and data aquisition, paticipation on meetings of the study	CHAGASICS Investigators
Silvana A. D.	Nishioka		PhD, MD	Department of Cardiology, Instituto do Coração (InCor), Hospital das Clínicas da Universidade de São Paulo	Sao Paulo, SP, Brazil	Follow-up and data aquisition, paticipation on meetings of the study	CHAGASICS Investigators
Thiago O.	Hueb		PhD, MD	Department of Cardiology, Instituto do Coração (InCor), Hospital das Clínicas da Universidade de São Paulo	Sao Paulo, SP, Brazil	Follow-up and data aquisition, paticipation on meetings of the study	CHAGASICS Investigators
Eduardo A.	Rocha		PhD, MD	Hospital Universitário Walter Cantideo - Universidade Federal do Ceará	Fortaleza, CE, Brazil	Recruitment, follow-up and data aquisition, paticipation on meetings of the study	CHAGASICS Investigators
Marcelo P. M.	Monteiro		MD	Hospital Universitário Walter Cantideo - Universidade Federal do Ceará	Fortaleza, CE, Brazil	Recruitment, follow-up and data aquisition, paticipation on meetings of the study	CHAGASICS Investigators
Andre	Schmidt		PhD, MD	Department of Cardiology, Faculdade de Medicina de Ribeirão Preto, Universidade de São Paulo	Ribeirao Preto, SP, Brazil	Recruitment, follow-up and data aquisition, paticipation on meetings of the study	CHAGASICS Investigators
Henrique T.	Moreira		PhD, MD	Department of Cardiology, Faculdade de Medicina de Ribeirão Preto, Universidade de São Paulo	Ribeirao Preto, SP, Brazil	Recruitment, follow-up and data aquisition, paticipation on meetings of the study	CHAGASICS Investigators
Luiz A. B.	Sá		PhD, MD	Hospital das Clinicas da Universidade Federal de Goias	Goiania, GO, Brazil	Recruitment, monitoring, device implantation and patient data acquisition, participation in study meetings	CHAGASICS Investigators
Enia L.	Coutinho		BSc	Department of Cardiology, Escola Paulista de Medicina (UNIFESP)	Sao Paulo, SP, Brazil	Recruitment, monitoring, patient data acquisition, participation in study meetings	CHAGASICS Investigators
Eduardo	Palmegiani		MD	Instituto de Moléstias Cardiovasculares - IMC	Sao Jose do Rio Preto, SP, Brazil	Recruitment, follow-up and data aquisition, paticipation on meetings of the study	CHAGASICS Investigators
Thiago B. C.	Megid		MD	Instituto de Moléstias Cardiovasculares - IMC	Sao Jose do Rio Preto, SP, Brazil	Recruitment, follow-up and data aquisition, paticipation on meetings of the study	CHAGASICS Investigators
Gustavo G.	Gomes		MD	Instituto de Cardiologia do Distrito Federal - ICDF	Brasilia, DF, Brazil	Recruitment, follow-up and data aquisition, paticipation on meetings of the study	CHAGASICS Investigators
Joubert A. P.	Marques		MD	Instituto de Cardiologia do Distrito Federal - ICDF	Brasilia, DF, Brazil	Recruitment, follow-up and data aquisition, paticipation on meetings of the study	CHAGASICS Investigators
Marcelo A.	Nakazone		PhD, MD	Hospital de Base da Fundacao Faculdade Regional de Medicina de Sao Jose do Rio Preto (HB/FUNFARME)	Sao Jose do Rio Preto, SP, Brazil	Recruitment, follow-up and data aquisition, paticipation on meetings of the study	CHAGASICS Investigators
Anis	Rassi	Jr	PhD, MD	Cardiology Division, Anis Rassi Hospital	Goiânia, GO, Brazil		Steering Committee until 2023
Otávio	Berwanger		PhD, MD	Research Institute HCor–Hospital do Coracao	São Paulo, Brazil		Steering Committee until 2019
