# Supplementary material for: Amiodarone or Implantable Cardioverter-Defibrillator in Chagas Cardiomyopathy: The CHAGASICS Randomized Clinical Trial
Source: JAMA Cardiol. 2024 Oct 2;9(12):1073–81. doi: 10.1001/jamacardio.2024.3169 (PMC11447631; doi:10.1001/jamacardio.2024.3169)
Supplement: Supplement 4. — Data Sharing Statement [file jamacardiol-e243169-s004.pdf]

Data Sharing Statement

Data
Data available: No

Additional Information
Explanation for why data not available: By law, in Brazil, patient data access for research must be previously approved by the ethics committee.
